# Distinct monocyte phenotypes are associated with peri-coronary inflammation in persons with and without HIV and without cardiovascular disease

**DOI:** 10.3389/fimmu.2026.1960785

**Published:** 2026-10-20

**Authors:** Victoria R. Stephens, Mian B. Hossain, Joshua Nordman, Michael L. Freeman, Jean Jeudy, Shana AB Burrowes, Marcela Araujo de Oliveira Santana, Sarah E. Mitchell, Sumanth D. Prabhu, Michael M. Lederman, Celestine N. Wanjalla, Nicholas Funderburg, Shashwatee Bagchi

**Affiliations:** 1 Division of Infectious Diseases, Vanderbilt University Medical Center, Nashville, TN, United States; 2 Department of Biostatistics, School of Community Health and Policy, Morgan State University, Baltimore, MD, United States; 3 Division of Infectious Diseases, Department of Medicine, Washington University School of Medicine, St. Louis, MO, United States; 4 Rustbelt Center for AIDS Research, Division of Infectious Diseases and HIV Medicine, Department of Medicine, Case Western Reserve University School of Medicine, Cleveland, OH, United States; 5 Center for Global Health and Diseases, Department of Pathology, Case Western Reserve University School of Medicine, Cleveland, OH, United States; 6 Section of Cardiothoracic Imaging, Department of Radiology, University of Maryland School of Medicine, Baltimore, MD, United States; 7 Section of Infectious Diseases, Department of Medicine, Boston Chobanian and Avedisian School of Medicine, Boston, MA, United States; 8 Department of Medicine, Washington University School of Medicine, St. Louis, MO, United States; 9 Department of Medicine, Institute of Human Virology, University of Maryland School of Medicine, Baltimore, MD, United States; 10 Division of Cardiology, Department of Medicine, Washington University School of Medicine, St. Louis, MO, United States; 11 Veteran’s Health Administration, Tennessee Valley Healthcare System, Nashville, TN, United States; 12 Division of Medical Laboratory Science, School of Health and Rehabilitation Sciences, The Ohio State University, Columbus, OH, United States

**Keywords:** cardiovascular disease, computed tomography, fat attenuation index, HIV, imaging, inflammation, monocytes, pericoronary adipose tissue

## Abstract

**Background and objective:**

Chronic inflammation in persons with (PWH) and without HIV (PWoH) is linked to cardiovascular disease (CVD). The fat attenuation index (FAI), a marker of peri-coronary inflammation, predicts CVD risk. The relationship between FAI and monocyte phenotypes remains unclear. This study aimed to evaluate the relationship between monocyte subsets—classical (CD16dim intermediate/inflammatory monocytes and CD16neg), intermediate, and non-classical— and surface markers of activation and chemokine expression, and FAI in a cohort of PWH and PWoH, and compare relative differences by HIV serostatus.

**Methods:**

In this cross-sectional of a prospective observational cohort study, 58 virologically suppressed PWH and 16 PWoH without known CVD underwent coronary computed tomography angiography to measure FAI. Peripheral blood monocyte subsets and activation/homing phenotypes were analyzed via flow cytometry. Multivariable regression models assessed correlations between monocyte markers and FAI in the LAD and RCA, with interaction terms testing effects of HIV serostatus.

**Results:**

Frequency and expression of CD40 were elevated in PWH, while HLA-DR expression was lower across all monocyte subsets compared to PWoH. CD38^+^ intermediate monocytes were linked to worsened LAD FAI. CD38^+^ non-classical monocytes were correlated with worsened RCA FAI. CCR2 expression on total classical, and CD16dim, and CD16neg monocytes correlated with worsened RCA and LAD FAI, while CX3CR1^+^ monocytes and CD40^+^ subsets correlated with improved RCA FAI in adjusted analyses.

**Conclusions:**

FAI demonstrates strong correlations with monocyte phenotypes with relationships influenced by HIV serostatus, highlighting activation markers and chemokine receptor profiles as plausible drivers of vascular inflammation and FAI as a tool for detecting early immune-mediated vascular inflammation.

## 1 Introduction

Cardiovascular disease (CVD) is the leading cause of mortality worldwide (1, 2), and is projected to rise substantially by 2050 (3). Hence, there is significant clinical interest in identifying blood and radiologic markers that may predict the onset of early disease and/or serve as targets for developing early interventional and preventive strategies. Until the development of the fat attenuation index (FAI), noninvasive quantification of subclinical coronary artery inflammation was not feasible. FAI measures compositional changes in perivascular fat to assess coronary inflammation using standard coronary computed tomography angiography (CCTA) (4), enabling the quantification of early-stage inflammation in the three major coronary arteries: the right coronary artery (RCA), the left anterior descending artery (LAD), and the left circumflex coronary artery (5). FAI has also been shown to predict major cardiac mortality and major adverse cardiovascular events (MACE), regardless of the presence of CVD risk factors or structural coronary artery disease (5, 6).

Persons with HIV (PWH) have a two-fold higher risk of developing CVD than persons without HIV (PWoH) (7). The elevated risk in PWH may be attributed in part to chronic inflammation and persistent immune activation (8–10). Monocyte infiltration and polarization play pivotal roles in regulating atherogenesis through the inflammation of perivascular adipose tissue and the formation, destabilization, and rupture of plaques, facilitated by the release of a variety of cytokines and chemokines (11–14).

Monocytes are traditionally differentiated based on expression of the lipopolysaccharide receptor CD14 and the FcyIII receptor CD16. Single-cell transcriptomic studies and reviews indicate that human monocytes form related but distinct populations shaped by subset-specific transcriptional networks and epigenetic programming (15–19). Regulon-based network inference distinguishes classical (CD14++CD16-) from nonclassical (CD14+CD16++) monocytes through different transcription factor–centered activity profiles (e.g., HMGB2/CREB5/FOSB-enriched programs in classical cells versus TCF7L2/POU2F2-associated programs in nonclassical cells), while intermediate (CD14++CD16+) monocytes show overlapping regulon activity consistent with a transitional state along a continuous trajectory inferred by pseudotime (15). In addition, trained-immunity paradigms demonstrate that prior stimulation can induce durable chromatin and metabolic alterations that reconfigure subsequent transcriptional responses and yield multiple trained monocyte states detectable at single-cell resolution, with related signatures observed in inflammatory disease settings (e.g., ulcerative colitis, sepsis, COVID-19) (16). Together, these data demonstrate that monocyte subsets are not solely CD14/CD16-defined phenotypes but transcriptionally and epigenetically programmed populations with specialized roles in inflammatory effector functions, migration, and tissue/vascular surveillance (17, 19) and that substantial heterogeneity can also exist within subsets across individuals and health/disease states (18).

In humans, three distinct monocyte subsets are conventionally defined based on expression of CD14, CD16, and chemokine receptors: classical (CD14++CD16-) monocytes; which express CCR2; intermediate (CD14++CD16+) monocytes, which express both CCR2 and CX3CR1 (and relatively more CCR5 than do classical monocytes); and nonclassical (CD14+CD16++) monocytes, which do not express CCR2 but typically express a high frequency of CX3CR1 among monocyte subsets (11, 20). Functionally, these subsets are increasingly recognized as nonredundant effectors across inflammatory and antiviral contexts: classical monocytes are broadly pro-inflammatory, with robust capacity to secrete soluble mediators and to differentiate into monocyte-derived dendritic cells, thereby contributing to the crosstalk between innate and adaptive immunity; intermediate monocytes are enriched for antigen presentation and have been linked to immune activation during HIV infection; and nonclassical monocytes are commonly associated with antiviral surveillance and response programs (19).

Advancements in flow cytometry have enabled the re-examination of the heterogeneity of various cellular subsets (21, 22), including the conventional blood-derived monocytes (23). Monocyte subsets have distinct functions, including cytokine production and antigen presentation (24–26). CD16-expressing monocyte subsets are often implicated as being more pro-inflammatory (27, 28), but less is known about their *in vivo* activities, and discrepancies exist regarding the association between CD16-expressing monocytes and atherosclerosis (29, 30).

Prior studies have shown the roles of monocyte subsets in atherosclerotic CVD (ASCVD) among PWoH (27, 30–33). Numbers of non-classical and intermediate monocytes have been shown to be elevated in PWH compared to those of PWoH, with higher levels in PWH correlating with increased viremia, T cell activation, and plasma IL-6 levels (34). Interestingly, the monocyte activation profile in uncontrolled HIV infection was similar to that of acute coronary syndrome (ACS) in PWoH (20). However, there is a paucity of studies evaluating monocyte subsets between PWoH and PWH, and none have evaluated the relationship between monocytes defined by this extended gating and subclinical measures of atherosclerosis using CCTA and measuring peri-coronary inflammation (FAI).

Our group has previously reported associations between peri-coronary inflammation, as assessed by FAI values, and markers of systemic inflammation and T lymphocyte activation in persons asymptomatic for ASVCD and found that certain inflammatory pathways were prominent and more robustly associated with FAI in PWH than among PWoH (35, 36). In this study, we evaluated the relationship between monocyte subsets—classical (including CD16dim intermediate/inflammatory monocytes and CD16neg), intermediate, and non-classical— and surface markers of activation and chemokine expression, and FAI in a cohort of PWH and PWoH, and compared differences by HIV serostatus. Finally, we explored correlations of specific monocyte phenotypes with the soluble inflammatory markers and circulating T cell phenotypes we have previously evaluated in these donors, to generate testable hypotheses for future mechanistic studies.

## 2 Methods

### 2.1 Study participants and procedures

Fifty-eight adult PWH virologically suppressed on ART for at least six months and 21 adult PWoH from a parent prospective observational study (four study groups, also including hepatitis C virus (HCV) mono-infected and HIV/HCV co-infected) that was conducted and IRB-approved (IRB# HP-00074189) at the University of Maryland (UMD) to assess coronary plaque changes with direct-acting antiviral treatment were included in the current study. We have previously described the inclusion and exclusion criteria of the parent observational study (35–38). Briefly, all participants in the current analysis were seronegative for hepatitis C virus (HCV), and PWoH were age- and sex-matched to PWH with otherwise identical inclusion/exclusion criteria and were recruited and enrolled from the same clinics. Participants with and without HIV were enrolled using identical eligibility criteria, with participants without HIV age- and sex-matched to those with HIV. We recruited both groups through the same sources, including participating clinics, ResearchMatch.org, and a university campus newsletter for faculty, staff, and students. HIV infection was defined by prior documentation of a confirmed reactive anti–HIV antibody test or a positive HIV antigen/antibody combination assay. We defined hepatitis C virus (HCV) infection as a confirmed reactive anti-HCV antibody test; all participants included in the present analysis were anti-HCV antibody–negative. History of atherosclerotic cardiovascular disease (ASCVD) was initially obtained by participant self-report and then verified by comprehensive review of electronic medical records from the University of Maryland Medical Center and outside institutions (including records from prior hospitalizations and emergency department visits), after participants provided written authorization for release of medical information; study eligibility was determined only after this verification was completed.

Major exclusion criteria included: presence of unstable coronary syndromes or symptoms suggestive of ACS; systolic blood pressure >160 mmHg; low-density lipoprotein cholesterol (LDL-c) >160 mg/dL; poorly controlled diabetes mellitus (defined as a need for anti-glycemic therapies and/or HbA1c >7%); hemoglobin <7g/dL; and creatinine clearance <45 mL/min. Participants were also excluded for self-reported or chart-documented active illicit drug use within the prior 3 months, cardiac conduction abnormalities in the absence of a pacemaker, left ventricular ejection fraction <40% at enrollment, severe or uncontrolled asthma, contraindications to β-blockers or sublingual nitroglycerin, or any contraindication to coronary computed tomography angiography (CCTA). Following baseline assessment of sociodemographic characteristics, medical history, physical examination, and blood collection, eligible participants underwent CCTA, typically within 1 week of enrollment (range, 5 days to 2 weeks).

For the primary analyses assessing monocyte subsets and chemokine receptor expression with FAI, and as described in our earlier reports, 5 PWoH with the most extreme body mass index (BMI) >40 were removed from the original sample to make the BMI between study groups more comparable, since BMI was found to have the strongest association with FAI in our cohort (37).

### 2.2 CCTA and measurement of FAI

CCTA was performed using a third-generation dual-source CT scanner (SOMATOM Force; Siemens Medical Solutions, Forchheim, Germany) from September 2017 to June 2021 and analyzed as we have described in a prior publication (37). Peri-coronary fat attenuation was measured around the proximal RCA and LAD and was defined as the mean attenuation of all adipose-containing voxels between −190 and −30 Hounsfield units obtained from the outer wall of the vessel within a radius equal to the vessel diameter. A total length of 40 mm from the proximal portion of the RCA and LAD was measured, and the proximal 10 mm of the RCA were excluded to limit artifacts from the aortic wall. FAI analysis was performed by two radiologists who were blinded to participant serostatus, and the senior radiologist performed quality control, supervision, and assurance of the analyses.

### 2.3 Flow cytometry analysis

Cryopreserved human peripheral blood mononuclear cells (PBMCs) from PWH and PWoH were thawed, washed twice in Hank’s Balanced Salt Solution (HBSS) containing 2% fetal bovine serum (FBS) and counted. Staining was performed by mixing pellets of 2x10 (6) cells with 50 µL antibody mixture at 4°C for 45 min, followed by washing twice in 0.5% FBS-HBSS. Antibodies were diluted in 0.5% FBS-HBSS, and the optimal dilution of each antibody had been determined beforehand. All antibodies were purchased from BioLegend (San Diego, CA). Stained cells were immediately analyzed by polychromatic flow cytometry using a FACSAria II flow cytometer (BD Biosciences). We subdivided the classical monocytes into two subsets based on the expression of CD16 (CD16neg vs. CD16dim intermediate/inflammatory monocytes). To test whether the CD16dim intermediate/inflammatory monocytes and CD16neg subpopulations presented distinct subpopulations within the classical monocyte population, an unsupervised principal component analysis (PCA) was performed based on the 11-color flow cytometry (FlowJo, FlowJo LLC. Ashland, OR) (39). To rule out variations among PWoH, PBMCs from five additional healthy donors were used for the monocyte subpopulation classification analyses. The boundary between these two provisional subpopulations was rigorously determined by a fluorescence-minus-one control for the CD16 staining. To assess the expression levels of each surface marker, we independently analyzed percent positivity (with negativity defined by staining with an isotype control antibody) and mean fluorescence intensity (MFI). This approach was necessary because some markers exhibited high positivity rates (>90%), which could compress the range of variation within subsets and potentially obscure statistically significant differences. Detailed information on the monocyte antibody panel and the flow cytometry gating strategy can be found in the **Supplementary Data**. Flow data were analyzed using FlowJo software, version 10 (FlowJo LLC).

### 2.4 Statistical analysis

We summarized and compared sociodemographic factors, behavioral indicators, clinical indicators, and mean FAI of both RCA and LAD between PWH and seronegative controls (PWoH). We restricted our analyses to the RCA and LAD because the original CRISP CT study found that the RCA and LAD FAI values predicted mortality and cardiac events (4), reflecting the spatially heterogeneous nature of atherosclerosis. Assumption of normality was checked for each continuous variable using Q-Q plots and Shapiro-Wilk test. For normally distributed continuous variables, an independent samples t-test was used to examine the difference between groups. For continuous variables that did not meet normality assumptions, a non-parametric Mann-Whitney U test was used to examine the difference between groups. For categorical variables, the Chi-square test or Fisher’s Exact test were used as appropriate to examine differences.

We analyzed the correlations (via Pearson’s or Spearman’s rank, as appropriate) and partial correlations of the percentages of monocyte subsets and their associated cell surface markers (both percentages and MFI) with mean FAI (RCA and LAD separately) in all study participants, controlling for age, sex, race, LDL-c, lipid-lowering medication use, and BMI. All covariates, except BMI, are presented as dichotomous variables, while BMI was presented as a continuous variable. For each model, we assessed the interaction between HIV serostatus and monocyte subset percentages and marker expression and adjusted for the confounders.

Additional analyses were conducted to assess correlations between soluble markers of inflammation and the monocyte subsets, as well as their associated surface markers. Simple Pearson’s correlations were examined in unadjusted and partial correlations were examined in adjusted analyses. Soluble markers assessed were IL-6, sTNF-R1, sTNF-R2, CCL2, CCL5, CX3CL1, CXCL10, sCD14, and sCD163. Similarly, simple Pearson’s correlations between monocyte subsets and surface markers and T cell subsets and surface markers were also explored. Since this was a hypothesis-generating study, we did not correct for multiple comparisons of these associations.

Statistical analysis was performed using STATA 18.0 (StataCorp, 2023) and the significance level was defined as *p*-values < 0.05.

## 3 Results

### 3.1 Characteristics of study participants

This study included 58 PWH and 16 PWoH, with no significant differences in age, sex, or traditional ASCVD risk factors, including BMI, antihypertensive use, and statin use (**Table 1**). While there was no difference in smoking between the groups, a higher proportion of PWH had a history of past alcohol use. PWH had significantly more education but lower income compared to PWoH. Peri-coronary inflammation, measured by FAI, was modestly but significantly greater in both the RCA (p=0.028) and LAD (p < 0.001) of PWoH compared to PWH.

**Table 1:** Sociodemographic and clinical characteristics of study population.

	People with HIV n=58 (73.4%)	People without HIV n=16 (26.6%)	*p-*value
Sociodemographic factors			
Sex at Birth, n (%)			
Female	27 (46.6%)	8 (50.0%)	0.807*
Age at enrollment (years) Mean ± SD	51.5 ± 9.37	51.6 ± 5.00	0.990^#^
Race, n (%)			0.018**
Black/African American	10 (17.5%)	8 (50.0%)	
White	47 (82.5%)	8 (50.0%)	
Education (n (%))			0.001*
High School Education or Less	12 (20.7%)	10 (66.7%)	
Post High School Education	40 (79.3%)	5 (33.3%)	
Income, n (%)			<0.001*
<$25,000	39 (67.2%)	2 (13.3%)	
Behavioral indicators			
Cigarette smoker			
Current	22 (37.9%)	4 (26.7%)	0.417*
Past	27 (51.9%)	4 (30.8%)	0.172*
Alcohol use			
Current	31 (53.5%)	13 (81.3%)	0.045*
Past	43 (84.3%)	5 (50.0%)	0.028**
Illicit drug use			
Current	3 (5.2%)	0 (0%)	1.000**
Past	16 (27.6%)	1 (6.3%)	0.097**
Clinical indicators			
Duration of HIV diagnosis years, Mean ± SD	17.8 ± 9.31		
ART duration, years, Median (Q1, Q3)	6 (2,8)		
CD4 cell count/mm3, Median (Q1, Q3)	682 (543, 1015)	1076 (1016, 1079)	0.123^¥^
Hepatitis B infected, n (%)	5 (8.6%)	0 (0%)	0.576**
CKD, GFR<60, n (%) ml/min/1.73m2	2 (3.6%)		
Family History CVD, n(%)	19 (32.8%)	6 (40.0%)	0.598*
Baseline ASCVD risk score, Median (Q1, Q3)	0.048 (0.022, 0.089)	0.044 (0.037, 0.088)	0.931^¥^
BMI, Mean ± SD	27.16 ± 6.10	29.13 ± 6.09	0.256^#^
Waist Hip Ratio, Mean ± SD	0.95 ± 0.08	0.92 ± 0.08	0.221^#^
Metabolic Syndrome, n(%)	13 (22.4%)	1 (6.7%)	0.274**
Antihypertensive use n (%)	21 (36.2%)	7 (43.8%)	0.582*
Statin use, n (%)	7 (12.1%)	4 (26.7%)	0.221**
Other lipid-lowering medication use, n(%)	8 (16.3%)	4 (33.3%)	0.229**
Systolic blood pressure mm Hg, Mean ± SD	125.2 ± 13.94	125.2 ± 13.69	0.989^#^
Total Cholesterol, mg/dL, Median (Q1, Q3)	183 (151, 203)	187 (164, 213)	0.780^¥^
HDL, mg/dL, Median (Q1, Q3)	50.5 (46, 66)	53 (38, 78)	0.795^¥^
LDL, mg/dL, Median (Q1, Q3)	102.5 (76, 123)	109 (94, 126)	0.517^¥^
Triglycerides, mg/dL, Median (Q1, Q3)	94.5 (69, 132)	84 (59, 107)	0.224^¥^
RCA Mean FAI, Mean ± SD	-85.80 ± 1.14	-85.00 ± 1.43	**0.028^#^**
LAD Mean FAI, Mean ± SD	-86.11 ± 1.00	-84.93 ± 1.29	**<0.001^#^**

ART, antiretroviral therapy, ASCVD, atherosclerotic cardiovascular disease, BMI, body mass index, CVD, cardiovascular disease, HDL, high-density lipoprotein, HIV, human immunodeficiency virus, LAD, Left Anterior Descending artery, LDL, low-density lipoprotein, RCA, Right Coronary Artery.

Bolded *p*-values represent statistically significant results.

*p-value using the Chi-Square test.

**p-value using the Fisher’s Exact test.

^#^*p*-value using the Independent samples t-test.

^¥^*p-*value using the Mann-Whitney U test.

^Ŧ^6 missing observations: 5 in HIV-infected and 1 in HIV-uninfected.

### 3.2 Monocyte subsets and phenotypes in PWH and PWoH

We characterized the subsets and phenotypes of circulating monocytes from all participants by flow cytometry using two comprehensive panels (**Supplementary Figure S1**; **Supplementary Table S1**). Throughout this study, MFIs are reported specifically for the positive populations within each marker. The frequencies of monocyte subsets did not differ between PWH and PWoH. However, across all subsets, CCR2 expression was lower in PWH than in PWoH (**Table 2**; **Supplementary Table S2**). The frequency of CCR5+ classical, CD16dim intermediate/inflammatory monocytes, and intermediate monocytes was also lower in PWH. The frequency and MFI of CD40 expression (a marker of activation) were higher in PWH compared to PWoH, whereas HLA-DR MFI (a potential marker of antigen-presenting capacity) was significantly lower in PWH across all subsets. Additionally, we observed a distinct segregation of CD16dim intermediate/inflammatory monocytes and CD16neg subpopulations among the classical monocytes in PWoH, illustrated by differing levels of expression of CCR5, CX3CR1, and CD163 (**Supplementary Figure S2**).

**Table 2:** Differences in the percentages of monocyte subsets and associated cell markers between persons with and without HIV.

Monocyte subsets and associated cell surface markers	Persons with HIV n=58 (78.4%) median(Q1,Q3)/mean ± SD	Persons without HIV n=16 (21.6%) median(Q1,Q3)/mean ± SD	*p*-value
Total classical Monocytes: CD16dim intermediate/inflammatory and CD16neg			
CCR2+ MFI	2504.5 (1197, 3491)	5573 (3637.5, 6061)	0.009^**^
CCR5+ (%)	1.62 ± 1.902	4.571 ± 9.025	0.022^*^
CD40+ (%)	63.50 ± 11.154	56.17 ± 9.061	0.018^*^
CD40+ MFI	594.5 (512, 711)	345.5 (272.5, 551.5)	<0.001^**^
HLA-DR+ MFI	816 (374, 1464)	2692.5 (1214.5, 3358)	<0.001^**^
CD16dim intermediate/inflammatory monocytes			
CCR2+ MFI	2464 (1133, 3244)	5422 (3475.5, 6059)	0.015^**^
CCR5+ (%)	1.89 ± 2.488	5.63 ± 10.886	0.018^*^
CD40+ (%)	72.45 ± 15.698	63.32 ± 12.340	0.035^*^
CD40+ MFI	717 (633, 892)	408.5 (321.5, 698.5)	<0.001^**^
HLA-DR+ (%)	83.19 ± 17.627	93.47 ± 6.680	0.026^*^
HLA-DR+ MFI	796.5 (313, 1470)	2772 (1258, 3417)	<0.001^**^
CD16neg monocytes			
CCR2+ MFI	3237.5 (1293, 4641)	6518.5 (4068, 7311.5)	0.015^**^
CD40+ MFI	367 (294, 484)	218.5 (188.5, 275)	<0.001^**^
HLA-DR+ MFI	856 (319, 1445)	2258.5 (1107, 3091)	<0.001^**^
Intermediate monocytes			
CCR2+ MFI	172.0 (108.0, 401.0)	627.0 (474.5, 686.5)	0.017^**^
CCR5+ (%)	4.99 ± 5.215	12.04 ± 12.169	<0.001^*^
CD40+ MFI	966 (753, 1221)	558 (461.5, 833.5)	<0.001^**^
HLA-DR+ MFI	2098.5 (828, 3437)	5654.5 (2386.5, 6981.5)	<0.001^**^
Non-classical monocytes			
CCR2+ MFI	60.8 (33.6, 134.0)	136.5 (93.35, 258.50)	0.016^**^
CD40+ (%)	64.19 ± 19.313	50.47 ± 15.960	0.011^**^
CD40+ MFI	676.5 (499, 841)	341 (196.5, 547.5)	<0.001^**^
HLA-DR+ MFI	906.5 (432, 1606)	2241.5 (1183, 3006.5)	0.002^**^

CCR2, C-C Motif Chemokine Receptor 2; CD, cluster of differentiation; DR, DR isotype of human leukocyte antigen; HIV, human immunodeficiency virus; MFI, mean fluorescent intensity.

*pvalue using Independent samples t-test for the means.

**pvalue using Mann-Whitney U test for the medians.

Bold *p*-values indicate significance.

### 3.3 Correlations of monocyte subsets and phenotypes with FAI in all participants

We next investigated the correlations between the mean FAI values of RCA and LAD and monocyte subsets (total classical, CD16dim intermediate/inflammatory monocytes, CD16neg, intermediate, and non-classical), further classified by surface marker expression. We report unadjusted and adjusted (**Table 3**; **Supplementary Table S3**) analyses, which controlled for age, sex, race, LDL-c, lipid-lowering medication use, and BMI. A positive correlation coefficient indicates that the monocyte feature is independently corelated with a worse mean FAI value (greater peri-coronary inflammation). In contrast, a negative coefficient indicates a better mean FAI value (less peri-coronary inflammation).

**Table 3:** Significant adjusted associations between monocyte subsets, associated cell surface markers, and FAI in all study participants.

All study participants (persons with and without HIV) n=74				
Monocyte subsets and associated cell surface markers	RCA Mean FAI		LAD mean FAI	
	^#^Partial correlation coefficient	*p-*value	^#^Partial correlation coefficient	*p-*value
Intermediate monocytes				
CD38+ (%)	0.130	0.372	0.289	0.034^*^
CD38+ MFI	0.138	0.344	0.317	0.020^*^
Non-classical monocytes				
CD38+ (%)	0.285	0.047^*^	0.196	0.156^*^
CD38+ MFI	0.046	0.751	0.325	0.017^*^
CD11b+ (%)	0.311	0.030^*^	0.226	0.100^*^
CD11b+ MFI	0.360	0.011^*^	0.186	0.179^*^

CD, cluster of differentiation; FAI, fat attenuation index; LAD, Left Anterior Descending artery; LDL-c, low density lipoprotein cholesterol; MFI, mean fluorescent intensity; RCA, Right Coronary Artery.

*Pearson’s Correlation Coefficient.

^#^Partial correlation coefficient after controlling for age, sex, race, LDL-c, lipid lowering medication use and BMI and p value – correlation coefficient (p value).

Bolded ***p***-values represent statistically significant results.

In adjusted analyses, CD38+ non-classical monocytes (β = 0.285; p = 0.047) and CD11b+ non-classical monocytes (β = 0.311; p = 0.030) were significantly correlated with worsened peri-coronary inflammation in the RCA (**Table 3**; **Supplementary Table S3**). Similar to unadjusted analyses, CD38+ intermediate monocytes were significantly correlated with LAD FAI (β = 0.289; p = 0.034).

### 3.4 Comparison of monocyte subsets and phenotypes with FAI in PWH and PWoH

We next examined the interactions between monocyte subsets and HIV serostatus on peri-coronary inflammation. A positive interaction value suggests that the relative difference in the mean FAI for each monocyte subset among PWH correlates more strongly with peri-coronary inflammation than in PWoH. Conversely, a negative interaction value suggests that the relative difference in mean FAI for a given monocyte subset among PWH correlates less strongly with peri-coronary inflammation than in PWoH. Overall, these analyses revealed numerous correlations with monocyte phenotypes and RCA and LAD FAI in both unadjusted (**Supplementary Table S4**) and adjusted analyses (**Table 4**; **Supplementary Table S5**).

**Table 4:** Significant adjusted associations between FAI and the interaction of HIV serostatus with monocyte subsets and associated surface markers (n=74).

Monocyte subsets and associated cell surface markers	RCA mean FAI marker x serostatus interaction		LAD mean FAI marker x serostatus interaction	
	Coefficient (95% CI)	*p*-value	Coefficient (95% CI)	*p*-value
Total classical monocytes (CD16dim intermediate/inflammatory and CD16neg)				
^¥^CCR2+ MFI × 10^-3^	0.369 (0.096, 0.642)	**0.009**	0.237 (0.061, 0.414)	**0.009**
^¥^CD40+ MFI × 10^-1^	-0.053 (-0.092, -0.015)	**0.007**	-0.004 (-0.037, 0.028)	0.782
CX3CR1+ (%)	-0.062 (-0.116, -0.008)	**0.025**	0.006 (-0.044, 0.056)	0.819
HLA-DR+ (%)	0.058 (-0.050, 0.166)	0.287	0.102 (0.008, 0.196)	**0.034**
^¥^ HLA-DR+ MFI × 10^-2^	0.065 (0.006, 0.123)	**0.032**	0.065 (0.011, 0.119)	**0.020**
^¥^CD11b+ MFI × 10^-3^	-0.332 (-0.630, -0.034)	**0.030**	0.137 (-0.038, 0.311)	0.122
CD16dim intermediate/inflammatory monocytes				
^¥^CCR2+ MFI × 10^-3^	0.362 (0.083, 0.641)	**0.012**	0.258 (0.056, 0.459)	**0.013**
CCR5+ (%)	0.356 (0.021, 0.690)	**0.038**	0.224 (-0.058, 0.506)	0.117
CD38+ (%)	0.193 (0.008, 0.378)	**0.041**	0.035 (-0.134, 0.204)	0.677
CD40+ (%)	-0.072 (-0.123, -0.021)	**0.006**	-0.026 (-0.076, 0.024)	0.295
^¥^CD40+ MFI × 10^-1^	-0.033 (-0.060, -0.007)	**0.014**	-0.003 (-0.027, 0.021)	0.781
CX3CR1+ (%)	-0.065 (-0.112, -0.019)	**0.007**	-0.003 (-0.048, 0.042)	0.895
^¥^ HLA-DR+ MFI × 10^-2^	0.056 (-0.001, 0.114)	0.055	0.058 (0.004, 0.111)	**0.034**
^¥^CD11b+ MFI × 10^-3^	-0.329 (-0.644, -0.014)	**0.041**	0.162 (-0.047, 0.371)	0.125
CD16neg monocytes				
^¥^CCR2+ MFI × 10^-3^	0.324 (0.083, 0.556)	**0.010**	0.178 (0.029, 0.328)	**0.020**
CD38+ (%)	0.152 (0.007, 0.297)	**0.041**	0.075 (-0.056, 0.206)	0.257
CX3CR1+ (%)	-0.037 (-0.063, -0.011)	**0.006**	-0.010 (-0.035, 0.015)	0.435
HLA-DR+ (%)	0.069 (-0.035, 0.174)	0.187	0.141 (0.057, 0.225)	**0.001**
^¥^ HLA-DR+ MFI × 10^-2^	0.081 (0.018, 0.144)	**0.013**	0.079 (0.021, 0.138)	**0.009**
Intermediate nonocytes				
CD40+ (%)	-0.096 (-0.161, -0.032)	**0.004**	-0.024 (-0.080, 0.031)	0.382
^¥^CD40+ MFI × 10^-1^	-0.032 (-0.050, -0.013)	**0.002**	-0.003 (-0.022, 0.016)	0.732
^¥^CD163+ MFI × 10^-2^	0.103 (0.022, 0.183)	**0.014**	0.034 (-0.043, 0.111)	0.377
Non-classical monocytes				
CD40+ (%)	-0.048 (-0.092, -0.004)	**0.032**	-0.011 (-0.055, 0.032)	0.601
^¥^CD40+ MFI × 10^-1^	-0.036 (-0.063, -0.010)	**0.008**	-0.003 (-0.027, 0.021)	0.816
^¥^ HLA-DR+ MFI × 10^-2^	0.073 (0.001, 0.144)	**0.046**	0.017 (-0.049, 0.084)	0.602

CCR2, C-C Motif Chemokine Receptor 2; CCR5, C-C chemokine receptor type 5; CD, cluster of differentiation; CX3CR1, C-X3-C Motif Chemokine Receptor 1; DR, DR isotype of human leukocyte antigen; FAI, fat attenuation index; LAD, Left anterior descending artery; LDL-c, low density lipoprotein cholesterol; MFI, mean fluorescent intensity; RCA, Right coronary artery.

^¥^Rescaled.

^#^Regression coefficient after adjusting for age, sex, race, LDL-c, lipid lowering medication use and BMI.

Bolded ***p***-values represent statistically significant results.

In adjusted analyses, CCR2 expression on monocytes showed significant correlations with increased peri-coronary inflammation in both the RCA and LAD in PWH as compared to PWoH. These correlations were observed across multiple monocyte subsets: total classical monocytes (β = 0.369, p = 0.009; β = 0.237, p = 0.009), CD16dim intermediate/inflammatory monocytes (β = 0.362, p = 0.012; β = 0.258, p = 0.013), and CD16neg monocytes (β = 0.324, p = 0.010; β = 0.178, p = 0.020) (**Table 4**; **Supplementary Table S5**). Additionally, specific monocyte phenotypes were positively correlated with worsened peri-coronary inflammation in the RCA among PWH: CCR5^+^ CD16dim intermediate/inflammatory monocytes (β = 0.356, p = 0.038), CD38^+^ CD16dim intermediate/inflammatory monocytes (β = 0.193, p = 0.041), and CD38^+^ CD16neg monocytes (β = 0.152, p = 0.041).

In contrast, several monocyte subsets and phenotypes were significantly correlated with improved peri-coronary inflammation in the RCA among PWH compared to PWoH. We found this relationship in adjusted analyses for: CX3CR1^+^ total classical monocytes (β = -0.062; p = 0.025), CX3CR1^+^ CD16dim intermediate/inflammatory monocytes (β = -0.065; p = 0.007), CX3CR1^+^ CD16neg monocytes (β = -0.037; p = 0.006), CD40^+^ CD16dim intermediate/inflammatory monocytes (β = -0.072; p = 0.006), CD40^+^ intermediate monocytes (β = -0.096; p = 0.004), and CD40^+^ non-classical monocytes (β = -0.048; p = 0.032) (**Table 4**; **Supplementary Table S5**).

### 3.5 Monocyte subset and chemokine receptor associations with soluble inflammatory markers in PWH and PWoH

Given that monocytes may affect CVD outcomes either directly or indirectly via cytokine release, we next examined correlations between monocyte subsets and phenotypes with soluble inflammatory markers (**Figure 1**; **Supplementary Tables S6, S7**).

**Figure 1:**
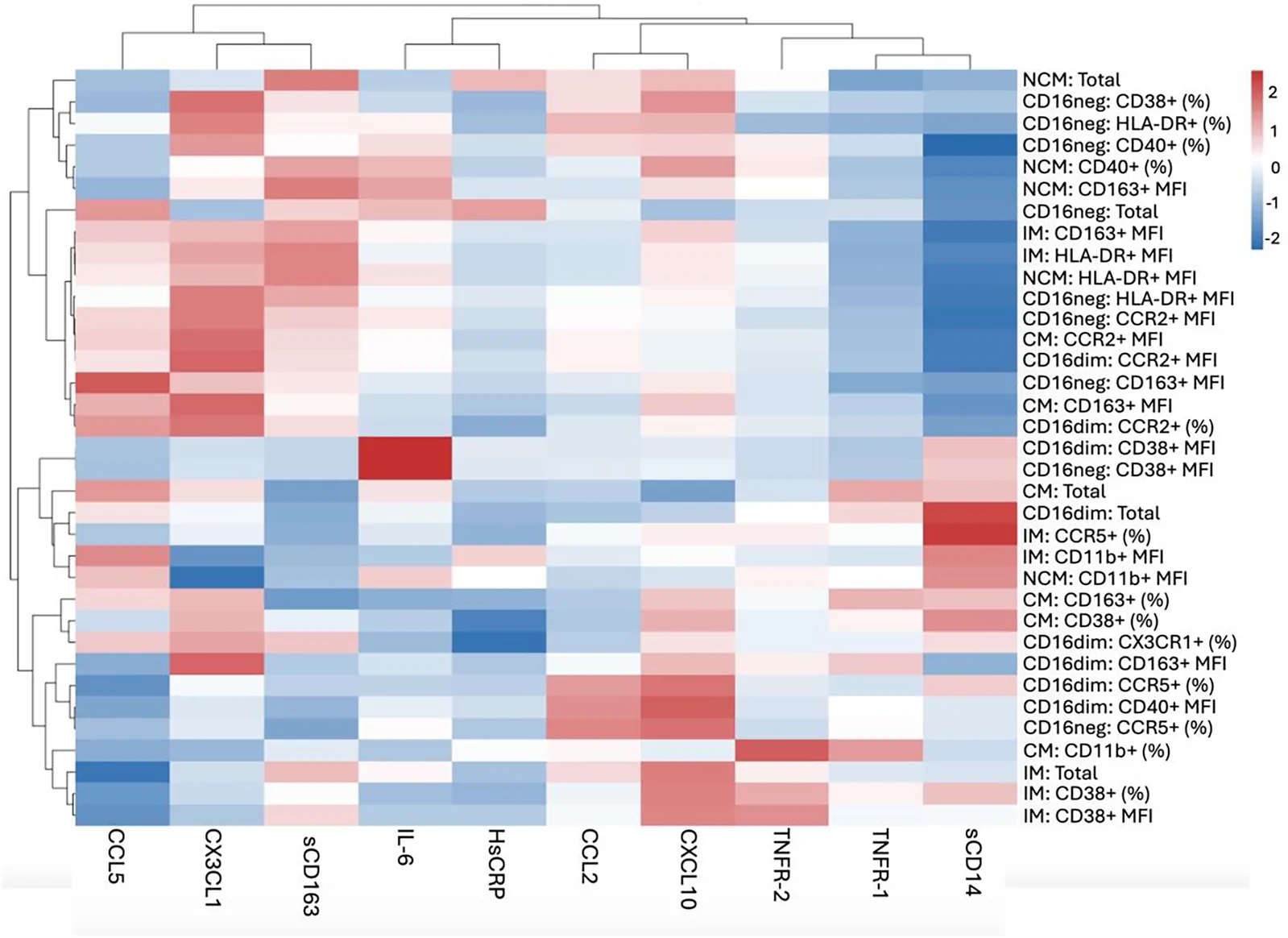
Significant associations between soluble markers and monocyte subset phenotypes adjusted for HIV serostatus. Clustered heatmap of statistically significant Pearson correlations between cellular markers and soluble markers. Heatmap tiles show Pearson correlation coefficients (r) for associations between cellular markers (rows) and soluble markers (columns). Correlation values were standardized using z-scores across each row. Colors indicate the direction and magnitude of r (red = positive, blue = negative, white ≈ 0). Rows and columns are hierarchically clustered using correlation distance (1 − r) to group markers with similar correlation profiles; dendrograms depict cluster structure.

After adjustment for HIV status, we found that sCD14 was negatively correlated with CCR2 expression intensity on classical monocytes, CD16dim intermediate/inflammatory monocytes and CD16neg monocytes but positively correlated with CCR5 expression on intermediate monocytes (**Figure 1**; **Supplementary Table S7**). In CD16neg monocytes, sCD14 was positively correlated with HLA-DR expression, and in intermediate monocytes, a positive correlation was observed with CD11b expression and a negative correlation with HLA-DR expression intensity. CXCL10 also demonstrated multiple correlations across subsets after adjustment for HIV status. Correlations were observed with CCR5 expression on CD16dim intermediate/inflammatory monocytes and CD16neg monocytes. CXCL10 was also correlated with CD40 and CD163 expression intensity on CD16dim intermediate/inflammatory monocytes. In CD16neg monocytes, CXCL10 was correlated with CD38 and CD40 expression on intermediate and non-classical monocytes, respectively. Correlations with CX3CL1 were more restricted, with correlations observed for CCR2 expression in classical monocytes and CD16dim intermediate/inflammatory monocytes. Taken together, the most consistent pattern that emerges is that sCD14 and CXCL10 remained the dominant soluble factors correlating with monocyte phenotypes, and sCD163 emerged after adjusting for HIV status, but mostly with intermediate and non-classical monocytes.

### 3.6 Analysis of T lymphocyte and monocyte subset interactions through cell surface marker correlations

We examined correlations between monocyte subsets and T lymphocyte subsets defined by cell surface markers (**Supplementary Figure S3**; **Supplementary Table S8**). HLA-DR+ classical monocytes exhibited negative correlations with CD57+ CX3CR1+ CD4+ T effector memory cells (TEM) and CD4+ T effector memory RA (TEMRA) subsets, as well as with PD-1+ CD4+ T central memory (TCM) and TEMRA cells. HLA-DR+ classical monocytes also uniquely displayed a positive and consistent correlation with CCR2+ CD4+ T cells across all subsets (TCM, TEM, and TEMRA).

Among intermediate monocytes, CD38+ intermediate monocytes positively correlated with CD38+ CD4+ T cells, HLA-DR+ and CD38+ HLA-DR+ CD4+ TCM and TEM subsets. These CD38+ intermediate monocytes also correlated with CD38+ CD8+ TEM and TEMRA as well as PD-1+ CD8+ TCM and TEM subsets. CD40+ intermediate monocytes showed positive correlations with HLA-DR+ and CD38+ HLA-DR+ CD4+ TCM and TEM. CD40+ intermediate monocytes were also positively correlated with CD38+ CD8+ TEM and TEMRA subsets.

Among non-classical monocytes, CD40+ non-classical monocytes positively correlated with CD38+ HLA-DR+ CD4+ TCM and TEM subsets. CD40+ non-classical monocytes were also positively correlated with CD57+ CX3CR1+ perforin/granzyme B+ CD4+ TEM and TEMRA cells.

CCR2+ classical monocytes were negatively correlated with CCR5+ CD4+ TCM, TEM, and TEMRA cells as well as CCR5+ CD8+ TEM and TEMRA subsets. In contrast, HLA-DR+ classical monocytes were positively correlated with CCR2+ CD8+ TCM and TEM cells. CCR2+ intermediate monocytes positively correlated with CD40+ intermediate monocytes. They also correlated with CD38+ and CD38+ HLA-DR+ CD4+ TCM and TEM subsets and HLA-DR+ CD4+ T cells across all subsets, highlighting their functional alignment with activated T cells. CCR5+ intermediate monocytes were negatively correlated with CD57+ CX3CR1+ CD8+ TCM and TEM subsets. Lastly, CCR5+ non-classical monocytes cells positively correlated with CX3CR1+ CD8+ TEM and TEMRA subsets.

## 4 Discussion

In this study, we demonstrate that monocyte activation phenotypes, rather than simple subset enumeration, are associated with peri-coronary inflammation as measured by FAI among asymptomatic individuals without prior known clinical ASCVD, with and without HIV. One of the major observations was that activated CD38^+^ intermediate and non-classical monocytes correlated with worsened FAI. In parallel, soluble activation markers sCD14 and CXCL10 (IP-10) showed the most consistent correlations with monocyte subsets. These data extend previous observations in both PWH and PWoH by linking activation of specific monocyte phenotypes to early peri-coronary inflammation, beyond systemic inflammation, detectable by noninvasive imaging. The integration of cell-surface activation phenotypes with soluble markers strengthens the concept that systemic immune activation is mirrored by vascular inflammation. Additionally, we report on CD40 expression on monocytes, not previously reported in PWH and atherosclerosis, as well as a subpopulation of classical monocytes (CD16dim intermediate/inflammatory monocytes) and raise the possibility of their role in peri-coronary inflammation, warranting further examination in future studies.

CD40/CD40L signaling is classically considered pro-inflammatory and pro-atherogenic (40–44), but we found that CD40 expression was associated with improved FAI in PWH despite increasing numbers of circulating CD40+ activated monocytes across all subsets. These findings suggest that increased circulating numbers of CD40^+^ activated monocytes may be engaged in controlling peri-coronary inflammation at the early stage of atherogenesis in PWH. It is possible this may be due to our measurement of the cell surface marker, as CD40L levels peak in late-stage atherosclerotic plaques when much of the plaque development has already occurred and CD40 is internalized (41). Our data suggests the possibility that CD40/CD40L axis may stage-specific roles in atherosclerosis, exerting both pro-inflammatory and regulatory roles in different contexts. To our knowledge, this is the first report of increased CD40 expression in monocytes of PWH associated with an early stage of atherosclerosis. Additional mechanistic analysis is needed to confirm the role of CD40+ monocytes in atherogenesis.

Chemokine receptor expression further distinguished the contributions of monocytes to vascular inflammation. CCR2 is a key pro-inflammatory chemokine receptor that mediates monocyte egress from the bone marrow and subsequent recruitment to inflamed tissues in response to its ligand CCL2 (MCP-1) (45, 46). By promoting trafficking of CCR2^+^ classical and intermediate monocytes to sites of endothelial activation, CCR2 signaling amplifies local cytokine production, macrophage accumulation, and vascular inflammatory remodeling—processes central to atherogenesis (47). In our study, CCR2 expression across subsets consistently correlated with worsened FAI, in line with prior studies (27, 48–50). Despite lower CCR2 MFI in PWH, CCR2^+^ monocytes retained a strong correlation with perivascular inflammation by FAI, underscoring the pathogenic potential of this subset, irrespective of receptor density (51). Conversely, our study found that CX3CR1 expression correlated with improved FAI in our cohort, while previously another study showed that an increase in levels of CX3CR1-expressing circulating monocytes were associated with increased carotid intima-media thickness (52). These and other data suggest that to the role of CX3CR1 in CVD may be stage-dependent (49, 53, 54). CCR5 was less broadly implicated but showed correlations with CD16dim intermediate/inflammatory monocytes, suggesting that this less-studied subset may have unique relevance in the early stages of atherosclerosis.

Our results also highlight heterogeneity within the classical monocytes. CD14+ CD16dim intermediate/inflammatory monocytes and CD14+ CD16neg monocytes exhibited differential expression of CCR5 and CX3CR1, as well as distinct correlations with inflammatory markers. CD16dim intermediate/inflammatory monocytes correlated with sCD14 and CXCL10, suggesting greater systemic activation and responsiveness to IFN-γ–driven inflammation (55). These data suggest that CD16dim intermediate/inflammatory monocytes may represent a functionally distinct population relevant to early vascular injury, though further phenotyping will be required to establish this phenomenon.

Non-classical monocytes, typically regarded as patrolling cells (20, 27), exhibited divergent correlations depending on their phenotype, with CD38^+^ non-classical monocytes being correlated with worsened FAI. In contrast, CD40^+^ non-classical monocytes correlated with lower FAI, particularly in PWH. Similarly, intermediate monocytes, long implicated in cardiovascular events (27, 29, 31, 56), were pro-inflammatory in our cohort, consistent with prior reports. These observations collectively align with the idea of a continuum of phenotypes with stage- and context-specific contributions to atherogenesis.

Our findings reveal a complex interplay between monocyte and T lymphocyte subsets, suggesting the possibility that specific monocyte populations may differentially regulate T cell activation, differentiation, and function. This highlights the potential immunomodulatory roles of monocyte subsets in shaping adaptive immune responses. Classical monocytes showed consistent and negative correlations with multiple T cell populations, with HLA-DR+ classical monocytes uniquely demonstrating positive correlations with activated CCR2+ CD4+ T cells. Intermediate monocytes emerged as key players in correlations with activated and inhibitory T cell markers, such as CD38, HLA-DR, and PD-1, across both CD4+ and CD8+ subsets. Non-classical monocytes demonstrated the fewest correlations overall, but their correlations with cytotoxic and migratory T cell subsets suggest a role in specialized immune functions. Finally, we observed decreased HLA-DR expression across all monocyte subsets in PWH compared to PWoH, consistent with impaired antigen presentation in HIV (57–59). Together with altered chemokine receptor expression, these findings emphasize that HIV infection may reshape monocyte function in ways that likely accelerate vascular injury. These findings extend prior observations of heightened monocyte activation in PWH by linking specific phenotypes to vascular inflammation, an early step in atherosclerosis.

This study is, to our knowledge, the first to examine the relationship between monocyte activation, chemokine receptor expression, and peri-coronary inflammation in PWH and PWoH using FAI. Our results indicate that monocyte activation markers (CD38, HLA-DR, CD40) expression, and chemokine receptor profiles (CCR2, CX3CR1, CCR5) may be correlates of early vascular inflammation. This is the first study to identify and characterize CD40^+^ monocytes and CD16dim intermediate/inflammatory monocytes in the context of PWH and atherosclerosis, suggesting their involvement in peri-coronary inflammation and highlighting the need for further research to elucidate their mechanistic roles. Notably, HIV status modified several of associations within this study, suggesting that immune dysregulation in PWH alters monocyte contributions to peri-coronary inflammation, even antecedent to structural disease. These findings highlight FAI as a potentially valuable tool to detect early immune-mediated vascular injury and to monitor interventions aimed at reducing inflammation and cardiovascular risk.

## 5 Study limitations

Our cross-sectional design precludes causal inference, and longitudinal studies are needed to establish whether monocyte phenotypes predict FAI progression. Our sample size, though sufficient to detect consistent patterns across subsets, limits the ability to identify subtler associations. Furthermore, we excluded five PWoH with BMIs greater than 40 to better match the BMI between study groups, since BMI was found to have the strongest association with FAI in our cohort (37), but further reducing our sample size. In addition, the original raw flow cytometry data files and analysis workspaces required to generate supplementary histograms, representative plots, or additional mean fluorescence intensity (MFI) visualizations are no longer available, precluding retrospective generation of these figures for the current study. We did not correct for multiple comparisons of these correlations as this was a hypothesis-generating study, but importantly, the analysis was conducted for each individual marker separately with each of the individual FAI endpoints. A key limitation is that we did not generate transcriptional or epigenetic profiles of monocyte subsets, and integrating single-cell transcriptomic and chromatin-based assays in future studies will be important to define the regulatory programs underlying the observed phenotypes and their links to FAI. Additionally,peripheral blood phenotyping may not fully reflect immune activity in the vascular wall or adipose tissue, underscoring the need for further studies to better understand the relationship between circulating immune markers and localized tissue inflammation. Future studies that correlate circulating and tissue monocyte populations with peri-coronary inflammation could elucidate the role of monocytes in vascular inflammation and early atherosclerosis. Furthermore, additional work is required to incorporate plaque composition, viral co-infections (HCV, CMV), and the effects of ART regimens on study outcomes. Including these measures in future analyses could provide valuable insights into the clinical utility of FAI as a complement to standard coronary computed tomography angiography (CCTA) for assessing cardiovascular risk, including in PWH.

## Data Availability

The original contributions presented in the study are included in the article/**Supplementary Material**. Further inquiries can be directed to the corresponding author.

## Glossary

ACS: Acute Coronary Syndrome
ART: Antiretroviral Therapy
ASCVD: Atherosclerotic Cardiovascular Disease
CAC: Coronary Artery Calcification
CCL: C-C Motif Chemokine Ligand
CCR: C-C Motif Chemokine Receptor
CCTA: Coronary Computed Tomography Angiography
CD: Cluster of differentiation
TCM: Central Memory T cell
CMV: Cytomegalovirus
CXCL: C-X-C motif ligand
CX3CL: C-X3-C motif ligand
CX3CR1: C-X3-C motif receptor 1
TEM: Effector Memory T cell
TEMRA: Terminally Differentiated Effector Memory or Effector Memory RA T cell
FAI: Fat attenuation index
FBS: Fetal bovine serum
HBSS: Hank’s balanced salt solution
HCV: Hepatitis C Virus
HLA: Human Leukocyte Antigens
HRP: High-risk plaque
hs-CRP: High sensitivity C-reactive protein
HU: Hounsfield units
IFN: Interferon
IL: Interleukin
IP-10: Interferon-inducible protein-10
LAD: Left anterior descending artery
LAP: Low attenuation plaque
LDL-C: Low-density lipoprotein-cholesterol
Lp(a): Lipoprotein (a)
MACE: Major adverse cardiovascular events
MFI: Mean fluorescent intensity
PBS: Phosphate buffered saline
PD1: Programmed cell death protein 1
PDL-1: Programmed cell death protein 1 ligand
PR: Positive remodeling
PBMC: Peripheral blood mononuclear cells
PWH: Persons with HIV
PWoH: Persons without HIV
RCA: Right coronary artery
REPRIEVE: Randomized Trial to Prevent Vascular Events in HIV
TEMRA: Terminally differentiated effector memory T cell
TNF: Tumor necrosis factor
TNFR: Tumor necrosis factor receptor
UMD: University of Maryland
