# Supplementary material for: Distinct monocyte phenotypes are associated with peri-coronary inflammation in persons with and without HIV and without cardiovascular disease
Source: Front Immunol. 2026 Oct 20;17:1960785. doi: 10.3389/fimmu.2026.1960785 (PMC13727482; doi:10.3389/fimmu.2026.1960785)
Supplement: Supplementary file 1 [file SupplementaryFile1.docx]

**Figure S1: Representative flow cytometry and gating strategies for all study participants (n=74)**

**________**

**Table S1: Flow cytometry antibody panel used for this study**

| Markers | Fluorochrome | Clone Identity | Catalog # (Biolegend) |
| --- | --- | --- | --- |
| CCR2 | PE Cy7 | K036C2 | 357212 |
| CCR5 | PE-Dazzle 594 | J418F1 | 359126 |
| CD38 | Brilliant Violet 605 | HIT2 | 303532 |
| CD40 | PerCP Cy5.5 | 5C3 | 334316 |
| CD163 | PE-Dazzle 594 | GHI/61 | 333624 |
| CX3CR1 | Brilliant Violet 421 | 2A9-1 | 341620 |
| HLA-DR | AlexaFluoro 700 | L243 | 307626 |
| CD11b | Brilliant Violet 510 | ICRF44 | 301334 |

_________

**Supplementary Table 2: Differences in percentages of monocyte subsets and associated cell surface markers between persons with and without HIV (n=74)**

**Supplementary Table 2a: Total Classical Monocytes (CD16dim and CD16neg)**

|  | **Persons with HIV**  **N=58 (78.4%)**  **Median(Q1,Q3)/Mean±SD** | **Persons without HIV**  **N= 16 (21.6%)**  **Median(Q1,Q3)/Mean±SD** | ***p*-value** |
| --- | --- | --- | --- |
| **Total** | 90.70±5.218 | 90.54±5.007 | 0.911^*^ |
| **CCR2+ (%)** | 94.87±4.084 | 94.71±2.667 | 0.880^*^ |
| **CCR2+ MFI** | 2504.5 (1197, 3491) | 5573 (3637.5, 6061) | **0.009^**^** |
| **CCR5+ (%)** | 1.62±1.902 | 4.571±9.025 | **0.022**^*^ |
| **CD38+ (%)** | 92.09±5.513 | 92.88±3.884 | 0.596^*^ |
| **CD38+ MFI** | 815.5 (675, 1001) | 829 (699.5, 1006) | 0.798^**^ |
| **CD40+ (%)** | 63.50±11.154 | 56.17±9.061 | **0.018**^*^ |
| **CD40+ MFI** | 594.5 (512, 711) | 345.5 (272.5, 551.5) | **<0.001**^**^ |
| **CD163+ (%)** | 55.62±26.919 | 49.82±22.967 | 0.435^*^ |
| **CD163+ MFI** | 1760.5 (1186, 2851) | 2145.5 (1537.5, 2923) | 0.458^**^ |
| **CX3CR1+ (%)** | 67.16±16.543 | 72.72±15.344 | 0.231^*^ |
| **CX3CR1+ MFI** | 1183 (853, 1476) | 1011 (917.5, 1217) | 0.529^**^ |
| **HLA-DR+ (%)** | 86.15±15.364 | 92.84±6.539 | 0.095^*^ |
| **HLA-DR+ MFI** | 816 (374, 1464) | 2692.5 (1214.5, 3358) | **<0.001**^**^ |
| **CD11b+ (%)** | 99.33±4.561 | 99.96±0.073 | 0.586^*^ |
| **CD11b+ MFI** | 13300 (9925, 18300) | 13350 (10498.5, 13850) | 0.524^**^ |

Notes: CCR2= C-C Motif Chemokine Receptor 2; CCR5= C-C chemokine receptor type 5; CD= cluster of differentiation; CX3CR1= C-X3-C Motif Chemokine Receptor 1; HLA-DR= DR isotype of human leukocyte antigen; HIV= human immunodeficiency virus; MFI= mean fluorescent intensity

* *p* value using Independent samples t-test for the means.

** *p* value using Mann-Whitney U test for the medians.

Bold *p*-values indicate significance.

**Supplementary Table 2b**: **CD16dim Monocytes**

|  | **Persons with HIV**  **N=58 (78.4%)**  **Median(Q1,Q3)/Mean±SD** | **Persons without HIV**  **N=16 (21.6%)**  **Median(Q1,Q3)/Mean±SD** | ***p*-value** |
| --- | --- | --- | --- |
| **Total** | 65.80±13.205 | 66.85±12.371 | 0.776^*^ |
| **CCR2+ (%)** | 93.54±4.966 | 93.76±2.938 | 0.867^*^ |
| **CCR2+ MFI** | 2464 (1133, 3244) | 5422 (3475.5, 6059) | **0.015^**^** |
| **CCR5+ (%)** | 1.89±2.488 | 5.63±10.886 | **0.018**^*^ |
| **CD38+ (%)** | 92.54±5.179 | 92.67±3.523 | 0.926^*^ |
| **CD38+ MFI** | 869 (720, 1075) | 835 (753, 989.5) | 0.870^**^ |
| **CD40+ (%)** | 72.45±15.698 | 63.32±12.340 | **0.035**^*^ |
| **CD40+ MFI** | 717 (633, 892) | 408.5 (321.5, 698.5) | **<0.001^**^** |
| **CD163+ (%)** | 62.70±23.277 | 67.12±15.176 | 0.476^*^ |
| **CD163+ MFI** | 1975.5 (1332, 3024) | 2924.5 (1843.5, 3369) | 0.136^**^ |
| **CX3CR1+ (%)** | 80.13±14.492 | 80.93±16.552 | 0.850^*^ |
| **CX3CR1+ MFI** | 1412 (1058, 1840) | 1303.5 (1125.5, 1557.5) | 0.537^**^ |
| **HLA-DR+ (%)** | 83.19±17.627 | 93.47±6.680 | **0.026**^*^ |
| **HLA-DR+ MFI** | 796.5 (313, 1470) | 2772 (1258, 3417) | **<0.001**^**^ |
| **CD11b+ (%)** | 99.53±2.794 | 99.88±0.238 | 0.624^*^ |
| **CD11b+ MFI** | 12800 (9617, 17700) | 12900 (10350, 13800) | 0.613^**^ |

Notes: CCR2= C-C Motif Chemokine Receptor 2; CCR5= C-C chemokine receptor type 5; CD= cluster of differentiation; CX3CR1= C-X3-C Motif Chemokine Receptor 1; HLA-DR= DR isotype of human leukocyte antigen; HIV= human immunodeficiency virus; MFI= mean fluorescent intensity

* *p* value using Independent samples t-test for the means.

** *p* value using Mann-Whitney U test for the medians.

Bold *p*-values indicate significance.

**Supplementary Table 2c: CD16neg Monocytes**

|  | **Persons with HIV**  **N=58 (78.4%)**  **Median(Q1,Q3)/Mean±SD** | **Persons without HIV**  **N=16 (21.6%)**  **Median(Q1,Q3)/Mean±SD** | ***p*-value** |
| --- | --- | --- | --- |
| **Total** | 19.39±11.046 | 18.10±8.902 | 0.669^*^ |
| **CCR2+ (%)** | 95.56±5.840 | 96.15±2.268 | 0.694^*^ |
| **CCR2+ MFI** | 3237.5 (1293, 4641) | 6518.5 (4068, 7311.5) | **0.015^**^** |
| **CCR5+ (%)** | 0.97±1.358 | 0.94±0.912 | 0.922^*^ |
| **CD38+ (%)** | 90.49±7.151 | 91.16±4.575 | 0.724^*^ |
| **CD38+ MFI** | 851 (666, 1033) | 812 (719, 926.5) | 0.906^**^ |
| **CD40+ (%)** | 38.75±20.063 | 30.02±12.491 | 0.104^*^ |
| **CD40+ MFI** | 367 (294, 484) | 218.5 (188.5, 275) | **<0.001**^**^ |
| **CD163+ (%)** | 21.33 (5.29, 44.66) | 23.739 (12.54, 44.30) | 0.713^**^ |
| **CD163+ MFI** | 799.5 (301, 1747) | 1009 (479, 1739) | 0.529^**^ |
| **CX3CR1+ (%)** | 34.35±21.874 | 37.31±28.019 | 0.655^*^ |
| **CX3CR1+ MFI** | 512.0 (312.0, 799.0) | 378.5 (166.7, 639.5) | 0.151^**^ |
| **HLA-DR+ (%)** | 83.87±16.821 | 91.61±6.691 | 0.077^*^ |
| **HLA-DR+ MFI** | 856 (319, 1445) | 2258.5 (1107, 3091) | **0.001**^**^ |
| **CD11b+ (%)** | 98.20±11.79 | 99.84±0.325 | 0.582^*^ |
| **CD11b+ MFI** | 12400 (9275, 17400) | 12150 (9801.5, 13900) | 0.733^**^ |

Notes: CCR2= C-C Motif Chemokine Receptor 2; CCR5= C-C chemokine receptor type 5; CD= cluster of differentiation; CX3CR1= C-X3-C Motif Chemokine Receptor 1; HLA-DR= DR isotype of human leukocyte antigen; HIV= human immunodeficiency virus; MFI= mean fluorescent intensity

* *p* value using Independent samples t-test for the means.

** *p* value using Mann-Whitney U test for the medians.

Bold *p*-values indicate significance.

**Supplementary Table 2d: Intermediate Monocytes**

|  | **People with HIV**  **N=58 (78.4%)**  **Median(Q1,Q3)/Mean±SD** | **People without HIV**  **N=16 (21.6%)**  **Median(Q1,Q3)/Mean±SD** | ***p*-value** |
| --- | --- | --- | --- |
| **Total** | 2.10±1.210 | 2.27±1.268 | 0.638^*^ |
| **CCR2+ (%)** | 19.07±11.94 | 19.33±12.624 | 0.941^*^ |
| **CCR2+ MFI** | 172.0 (108.0, 401.0) | 627.0 (474.5, 686.5) | **0.017^**^** |
| **CCR5+ (%)** | 4.99±5.215 | 12.04±12.169 | **<0.001^*^** |
| **CD38+ (%)** | 31.44±19.813 | 32.86±16.436 | 0.793^*^ |
| **CD38+ MFI** | 175.5 (106, 243) | 212.5 (152.5, 274) | 0.176^**^ |
| **CD40+ (%)** | 81.77±11.318 | 75.84±11.685 | 0.070^*^ |
| **CD40+ MFI** | 966 (753, 1221) | 558 (461.5, 833.5) | **<0.001^*^**^*^ |
| **CD163+ (%)** | 65.34±17.618 | 61.91±18.031 | 0.495^*^ |
| **CD163+ MFI** | 2111 (1616, 2601) | 3025 (2173.5, 3279) | 0.064^**^ |
| **CX3CR1+ (%)** | 91.50±12.411 | 95.47±5.702 | 0.220^*^ |
| **CX3CR1+ MFI** | 3382 (2594, 4179) | 3771.5 (2980.5, 4676) | 0.189^**^ |
| **HLA-DR+ (%)** | 96.61±8.356 | 98.30±2.255 | 0.427^*^ |
| **HLA-DR+ MFI** | 2098.5 (828, 3437) | 5654.5 (2386.5, 6981.5) | **<0.001^*^**^*^ |
| **CD11b+ (%)** | 97.12±4.744 | 97.89±2.927 | 0.536^*^ |
| **CD11b+ MFI** | 6212.5 (4184, 9295) | 6026 (5230.5, 7195.5) | 0.581^**^ |

Notes: CCR2= C-C Motif Chemokine Receptor 2; CCR5= C-C chemokine receptor type 5; CD= cluster of differentiation; CX3CR1= C-X3-C Motif Chemokine Receptor 1; HLA-DR= DR isotype of human leukocyte antigen; HIV= human immunodeficiency virus; MFI= mean fluorescent intensity

* *p* value using Independent samples t-test for the means.

** *p* value using Mann-Whitney U test for the medians.

Bold *p*-values indicate significance.

**Supplementary Table 2e: Non-Classical Monocytes**

|  | **People with HIV**  **N=58 (78.4%)**  **Median(Q1,Q3)/Mean±SD** | **People without HIV**  **N=16 (21.6%)**  **Median(Q1,Q3)/Mean±SD** | ***p*-value** |
| --- | --- | --- | --- |
| **Total** | 7.30±4.553 | 7.20±4.423 | 0.937^*^ |
| **CCR2+ (%)** | 3.50±12.158 | 1.67±2.239 | 0.554^*^ |
| **CCR2+ MFI** | 60.8 (33.6, 134.0) | 136.5 (93.35, 258.50) | **0.016^**^** |
| **CCR5+ (%)** | 0.49 (0.24, 1.32) | 0.65 (0.31, 2.52) | 0.248^**^ |
| **CD38+ (%)** | 10.13±13.351 | 8.77±8.215 | 0.702^*^ |
| **CD38+ MFI** | 66.55 (37.0, 119) | 63.05 (38.4, 118.5) | 0.906^**^ |
| **CD40+ (%)** | 64.19±19.313 | 50.47±15.960 | **0.011^*^** |
| **CD40+ MFI** | 676.5 (499, 841) | 341 (196.5, 547.5) | **<0.001^*^**^*^ |
| **CD163+ (%)** | 29.84±15.750 | 26.827±11.932 | 0.480^*^ |
| **CD163+ MFI** | 1082 (808, 1302) | 1108 (861.5, 1711.5) | 0.318^**^ |
| **CX3CR1+ (%)** | 89.570±17.118 | 94.27±7.379 | 0.290^*^ |
| **CX3CR1+ MFI** | 3161 (2489, 4054) | 4176 (2901.5, 5405.5) | 0.090^**^ |
| **HLA-DR+ (%)** | 89.67±19.436 | 95.200±8.675 | 0.274^*^ |
| **HLA-DR+ MFI** | 906.5 (432, 1606) | 2241.5 (1183, 3006.5) | **0.002^*^**^*^ |
| **CD11b+ (%)** | 72.16±17.462 | 74.06±15.012 | 0.693^*^ |
| **CD11b+ MFI** | 1370 (796, 2230) | 1050.5 (850, 1422) | 0.217^**^ |

Notes: CCR2= C-C Motif Chemokine Receptor 2; CCR5= C-C chemokine receptor type 5; CD= cluster of differentiation; CX3CR1= C-X3-C Motif Chemokine Receptor 1; HLA-DR= DR isotype of human leukocyte antigen; HIV= human immunodeficiency virus; MFI= mean fluorescent intensity

* *p* value using Independent samples t-test for the means.

** *p* value using Mann-Whitney U test for the medians.

Bold *p*-values indicate significance.

**__________**

**Figure S2: Reclassification of Circulating Monocytes in all study participants (n = 74)**

**________**

**Supplementary Tables 3: Correlations and partial correlations between monocyte subsets and associated cell surface markers and fat attenuation index among all study participants (n=74)**

**Supplementary Table 3a: Total Classical Monocytes (CD16dim and CD16neg)**

| **All Study Participants (Persons with and without HIV) N=74** | | | | |
| --- | --- | --- | --- | --- |
|  | **RCA Mean FAI** | | **LAD Mean FAI** | |
|  | **Correlation Coefficient**  **(*p*-value)** | **^#^Partial Correlation Coefficient (*p*-value)** | **Correlation Coefficient**  **(*p*-value)** | **^#^Partial Correlation Coefficient (*p*-value)** |
| **Total** | -0.060 (0.625)^*^ | -0.072 (0.621)^*^ | -0.171 (0.145)^*^ | -0.220 (0.110)^*^ |
| **CCR2+ (%)** | -0.204 (0.095)^*^ | 0.003 (0.983)^*^ | -0.158 (0.179)^*^ | -0.045 (0.745)^*^ |
| **CCR2+ MFI** | -0.018 (0.883)^*^ | 0.105 (0.471)^*^ | -0.098 (0.408)^*^ | -0.073 (0.598)^*^ |
| **CCR5+ (%)** | 0.004 (0.974)^*^ | -0.059 (0.688)^*^ | 0.195 (0.095)^*^ | 0.063 (0.651)^*^ |
| **CD38+ (%)** | 0.043 (0.702)^*^ | 0.094 (0.522)^*^ | 0.026 (0.824)^*^ | 0.074 (0.597)^*^ |
| **CD38+ MFI** | 0.122 (0.323)^*^ | 0.212 (0.143)^*^ | -0.066 (0.576)^*^ | -0.052 (0.709)^*^ |
| **CD40+ (%)** | -0.063 (0.607)^*^ | 0.028 (0.850)^*^ | -0.069 (0.559)^*^ | -0.002 (0.986)^*^ |
| **CD40+ MFI** | -0.061 (0.619)^*^ | -0.054 (0.710)^*^ | -0.182 (0.122)^*^ | -0.178 (0.198)^*^ |
| **CD163+ (%)** | 0.063 (0.612)^*^ | -0.068 (0.645)^*^ | 0.043 (0.715)^*^ | -0.032 (0.821)^*^ |
| **CD163+ MFI** | 0.029 (0.816)^*^ | 0.039 (0.791)^*^ | -0.164 (0.164)^*^ | -0.133 (0.336)^*^ |
| **CX3CR1+ (%)** | 0.117 (0.340)^*^ | 0.178 (0.222)^*^ | 0.026 (0.829)^*^ | 0.004 (0.976)^*^ |
| **CX3CR1+ MFI** | 0.032 (0.794)^*^ | 0.017 (0.907)^*^ | -0.064 (0.591)^*^ | -0.098 (0.480)^*^ |
| **HLA-DR+ (%)** | 0.069 (0.579)^*^ | 0.047 (0.747)^*^ | 0.054 (0.645)^*^ | 0.064 (0.644)^*^ |
| **HLA-DR+ MFI** | 0.069 (0.576)^*^ | 0.034 (0.817)^*^ | 0.168 (0.153)^*^ | 0.137 (0.322)^*^ |
| **CD11b+ (%)** | -0.185 (0.132)^*^ | -0.111 (0.446)^*^ | 0.094 (0.424)^*^ | 0.079 (0.571)^*^ |
| **CD11b+ MFI** | 0.109 (0.378)^*^ | 0.105 (0.473)^*^ | -0.129 (0.274)^*^ | -0.171 (0.217)^*^ |

Notes: BMI= body mass index; CCR2= C-C Motif Chemokine Receptor 2; CCR5= C-C chemokine receptor type 5; CD= cluster of differentiation; CX3CR1= C-X3-C Motif Chemokine Receptor 1; DR= DR isotype of human leukocyte antigen; FAI= fat attenuation index; LAD= Left Anterior Descending artery; LDL-c= low density lipoprotein cholesterol; MFI=mean fluorescent intensity; RCA=Right Coronary Artery

* Pearson’s Correlation Coefficient. ** Spearman’s Rank Correlation Coefficient

\# partial correlation coefficient after controlling for age, sex, race, LDL-c, lipid lowering medication use and BMI and p value

**Supplementary Table 3b: CD16dim Monocytes**

| **All Study Participants (Persons with and without) N=74** | | | | |
| --- | --- | --- | --- | --- |
|  | **RCA Mean FAI** | | **LAD Mean FAI** | |
|  | **Correlation Coefficient**  **(*p*-value)** | **^#^Partial Correlation Coefficient (*p*-value)** | **Correlation Coefficient**  **(*p*-value)** | **^#^Partial Correlation Coefficient (*p*-value)** |
| **Total** | 0.127 (0.302)^*^ | 0.233 (0.107)^*^ | 0.069 (0.561)^*^ | 0.116 (0.403)^*^ |
| **CCR2+ (%)** | -0.194 (0.114)^*^ | -0.073 (0.616)^*^ | -0.082 (0.488)^*^ | -0.021 (0.879)^*^ |
| **CCR2+ MFI** | -0.025 (0.842)^*^ | 0.052 (0.720)^*^ | -0.100 (0.398)^*^ | -0.125 (0.368)^*^ |
| **CCR5+ (%)** | 0.067 (0.586)^*^ | -0.024 (0.872)^*^ | **0.229 (0.050)**^*^ | -0.002 (0.989)^*^ |
| **CD38+ (%)** | 0.024 (0.846)^*^ | 0.024 (0.871)^*^ | 0.056 (0.637)^*^ | 0.121 (0.384)^*^ |
| **CD38+ MFI** | -0.024 (0.847)^*^ | 0.003 (0.985)^*^ | -0.058 (0.621)^*^ | -0.030 (0.832)^*^ |
| **CD40+ (%)** | -0.161 (0.189)^*^ | -0.146 (0.318)^*^ | -0.071 (0.548)^*^ | -0.039 (0.782)^*^ |
| **CD40+ MFI** | 0.132 (0.283)^*^ | 0.158 (0.279)^*^ | 0.006 (0.959)^*^ | -0.098 (0.481)^*^ |
| **CD163+ (%)** | 0.046 (0.708)^*^ | -0.082 (0.575)^*^ | 0.014 (0.903)^*^ | -0.077 (0.578)^*^ |
| **CD163+ MFI** | -0.010 (0.933)^*^ | -0.169 (0.246)^*^ | -0.154 (0.191)^*^ | -0.115 (0.409)^*^ |
| **CX3CR1+ (%)** | -0.094 (0.448)^*^ | -0.152 (0.297)^*^ | -0.076 (0.518)^*^ | -0.067 (0.629)^*^ |
| **CX3CR1+ MFI** | -0.041 (0.742)^*^ | -0.123 (0.400)^*^ | -0.124 (0.292)^*^ | -0.163 (0.239)^*^ |
| **DR+ (%)** | 0.030 (0.809)^*^ | -0.014 (0.924)^*^ | 0.043 (0.717)^*^ | 0.061 (0.662)^*^ |
| **DR+ MFI** | 0.054 (0.662)^*^ | -0.018 (0.903)^*^ | 0.177 (0.131)^*^ | 0.108 (0.437)^*^ |
| **CD11b+ (%)** | 0.101 (0.414)^*^ | 0.241 (0.096)^*^ | 0.037 (0.758)^*^ | 0.222 (0.106)^*^ |
| **CD11b+ MFI** | 0.139 (0.260)^*^ | 0.148 (0.311)^*^ | -0.116 (0.327)^*^ | -0.160 (0.249)^*^ |

Notes: BMI= body mass index; CCR2= C-C Motif Chemokine Receptor 2; CCR5= C-C chemokine receptor type 5; CD= cluster of differentiation; CX3CR1= C-X3-C Motif Chemokine Receptor 1; HLA-DR= DR isotype of human leukocyte antigen ; FAI= fat attenuation index; LAD= Left Anterior Descending artery; LDL-c= low density lipoprotein cholesterol; MFI=mean fluorescent intensity; RCA=Right Coronary Artery

* Pearson’s Correlation Coefficient. ** Spearman’s Rank Correlation Coefficient

\# partial correlation coefficient after controlling for age, sex, race, LDL-c, lipid lowering medication use and BMI

**Supplementary Table 3c: CD16neg Monocytes**

| **All Study Participants (Persons with and without HIV) N=74** | | | | |
| --- | --- | --- | --- | --- |
|  | **RCA Mean FAI** | | **LAD Mean FAI** | |
|  | **Correlation Coefficient**  **(*p*-value)** | **^#^Partial Correlation Coefficient (*p*-value)** | **Correlation Coefficient**  **(*p*-value)** | **^#^Partial Correlation Coefficient (*p*-value)** |
| **Total** | -0.186 (0.130)^*^ | -0.208 (0.151)^*^ | -0.167 (0.155)^*^ | -0.208 (0.132)^*^ |
| **CCR2+ (%)** | -0.080 (0.515)^*^ | 0.014 (0.922)^*^ | -0.089 (0.454)^*^ | -0.051 (0.715)^*^ |
| **CCR2+ MFI** | -0.029 (0.814)^*^ | 0.032 (0.827)^*^ | -0.123 (0.298)^*^ | -0.126 (0.362)^*^ |
| **CCR5+ (%)** | 0.026 (0.831)^*^ | 0.061 (0.675)^*^ | -0.076 (0.518)^*^ | -0.146 (0.292)^*^ |
| **CD38+ (%)** | -0.017 (0.890)^*^ | 0.012 (0.932)^*^ | -0.006 (0.961)^*^ | 0.076 (0.583)^*^ |
| **CD38+ MFI** | -0.023 (0.854)^*^ | 0.005 (0.973)^*^ | -0.067 (0.572)^*^ | -0.029 (0.832)^*^ |
| **CD40+ (%)** | -0.060 (0.624)^*^ | -0.127 (0.383)^*^ | -0.192 (0.101)^*^ | -0.148 (0.284)^*^ |
| **CD40+ MFI** | -0.144 (0.243)^*^ | -0.131 (0.370)^*^ | **-0.326 (0.005)**^*^ | -0.231 (0.093)^*^ |
| **CD163+ (%)** | -0.019 (0.877)^*^ | -0.107 (0.466)^*^ | -0.066 (0.578)^*^ | -0.112 (0.418)^*^ |
| **CD163+ MFI** | -0.017 (0.891)^**^ | 0.019 (0.895)^*^ | -0.043 (0.715)^**^ | -0.129 (0.352)^*^ |
| **CX3CR1+ (%)** | 0.040 (0.744)^*^ | 0.093 (0.526)^*^ | -0.017 (0.883)^*^ | -0.125 (0.369)^*^ |
| **CX3CR1+ MFI** | -0.019 (0.876)^**^ | -0.051 (0.726)^*^ | -0.210 (0.073)^**^ | -0.191 (0.167)^*^ |
| **HLA-DR+ (%)** | 0.002 (0.985)^*^ | -0.044 (0.762)^*^ | -0.036 (0.764)^*^ | -0.020 (0.887)^*^ |
| **HLA-DR+ MFI** | 0.050 (0.685)^*^ | -0.005 (0.970)^*^ | 0.124 (0.293)^*^ | 0.089 (0.521)^*^ |
| **CD11b+ (%)** | 0.138 (0.264)^*^ | 0.032 (0.829)^*^ | 0.105 (0.374)^*^ | 0.013 (0.926)^*^ |
| **CD11b+ MFI** | 0.234 (0.054)^*^ | 0.169 (0.245)^*^ | -0.030 (0.797)^*^ | -0.056 (0.896)^*^ |

Notes: BMI= body mass index; CCR2= C-C Motif Chemokine Receptor 2; CCR5= C-C chemokine receptor type 5; CD= cluster of differentiation; CX3CR1= C-X3-C Motif Chemokine Receptor 1; HLA-DR= DR isotype of human leukocyte antigen; FAI= fat attenuation index; LAD= Left Anterior Descending artery; LDL-c= low density lipoprotein cholesterol; MFI=mean fluorescent intensity; RCA=Right Coronary Artery

* Pearson’s Correlation Coefficient. ** Spearman’s Rank Correlation Coefficient

\# partial correlation coefficient after controlling for age, sex, race, LDL-c, lipid lowering medication use and BMI

**Supplementary Table 3d: Intermediate Monocytes**

| **All Study Participants (Persons with and without HIV) N=74** | | | | |
| --- | --- | --- | --- | --- |
|  | **RCA Mean FAI** | | **LAD Mean FAI** | |
|  | **Correlation Coefficient**  **(*p*-value)** | **^#^Partial Correlation Coefficient (*p*-value)** | **Correlation Coefficient**  **(*p*-value)** | **^#^Partial Correlation Coefficient (*p*-value)** |
| **Total** | 0.056 (0.650)^*^ | 0.174 (0.231)^*^ | 0.119 (0.315)^*^ | 0.190 (0.168)^*^ |
| **CCR2+ (%)** | 0.003 (0.980)^*^ | 0.015 (0.921)^*^ | 0.088 (0.458)^*^ | 0.161 (0.244)^*^ |
| **CCR2+ MFI** | 0.012 (0.922)^**^ | 0.141 (0.333)^*^ | 0.027 (0.817)^**^ | 0.054 (0.696)^*^ |
| **CCR5+ (%)** | 0.092 (0.457)^*^ | 0.067 (0.646)^*^ | **0.252 (0.031)**^*^ | 0.234 (0.088)^*^ |
| **CD38+ (%)** | 0.112 (0.362)^*^ | 0.130 (0.372)^*^ | **0.295 (0.011)**^*^ | **0.289 (0.034)^*^** |
| **CD38+ MFI** | 0.161 (0.189)^*^ | 0.138 (0.344)^*^ | **0.294 (0.011)**^*^ | **0.317 (0.020)^*^** |
| **CD40+ (%)** | -0.070 (0.569)^*^ | 0.162 (0.267)^*^ | 0.051 (0.667)^*^ | 0.190 (0.169)^*^ |
| **CD40+ MFI** | -0.050 (0.683)^*^ | 0.045 (0.757)^*^ | -0.018 (0.882)^*^ | 0.099 (0.476)^*^ |
| **CD163+ (%)** | 0.006 (0.960)^*^ | -0.051 (0.726)^*^ | 0.036 (0.760)^*^ | 0.095 (0.496)^*^ |
| **CD163+ MFI** | -0.033 (0.789)^*^ | -0.022 (0.879)^*^ | -0.128 (0.277)^*^ | -0.111 (0.423)^*^ |
| **CX3CR1+ (%)** | -0.083 (0.503)^*^ | -0.115 (0.430)^*^ | 0.014 (0.903)^*^ | 0.056 (0.688)^*^ |
| **CX3CR1+ MFI** | 0.164 (0.183)^*^ | 0.123 (0.400)^*^ | 0.134 (0.254)^*^ | 0.004 (0.977)^*^ |
| **HLA-DR+ (%)** | 0.097 (0.434)^*^ | 0.215 (0.138)^*^ | 0.128 (0.277)^*^ | 0.190 (0.170)^*^ |
| **HLA-DR+ MFI** | 0.014 (0.910)^*^ | -0.038 (0.795)^*^ | 0.189 (0.108)^*^ | 0.181 (0.191)^*^ |
| **CD11b+ (%)** | 0.044 (0.724)^*^ | 0.173 (0.235)^*^ | 0.177 (0.132)^*^ | 0.218 (0.113)^*^ |
| **CD11b+ MFI** | 0.124 (0.312)^*^ | 0.016 (0.914)^*^ | 0.050 (0.673)^*^ | -0.027 (0.848)^*^ |

Notes: BMI= body mass index; CCR2= C-C Motif Chemokine Receptor 2; CCR5= C-C chemokine receptor type 5; CD= cluster of differentiation; CX3CR1= C-X3-C Motif Chemokine Receptor 1; HLA-DR= DR isotype of human leukocyte antigen ; FAI= fat attenuation index; LAD= Left Anterior Descending artery; LDL-c= low density lipoprotein cholesterol; MFI=mean fluorescent intensity; RCA=Right Coronary Artery

* Pearson’s Correlation Coefficient. ** Spearman’s Rank Correlation Coefficient

\# partial correlation coefficient after controlling for age, sex, race, LDL-c, lipid lowering medication use and BMI

**Supplementary Table 3e: Non-Classical Monocytes**

| **All Study Participants (Persons with and without HIV) N=74** | | | | |
| --- | --- | --- | --- | --- |
|  | **RCA Mean FAI** | | **LAD Mean FAI** | |
|  | **Correlation Coefficient**  **(*p*-value)** | **^#^Partial Correlation Coefficient (*p*-value)** | **Correlation Coefficient**  **(*p*-value)** | **^#^Partial Correlation Coefficient (*p*-value)** |
| **Total** | 0.036 (0.771)^*^ | 0.039 (0.789)^*^ | 0.147 (0.211)^*^ | 0.202 (0.144)^*^ |
| **CCR2+ (%)** | -0.091 (0.459)^*^ | -0.044 (0.762)^*^ | -0.051 (0.667)^*^ | -0.051 (0.715)^*^ |
| **CCR2+ MFI** | 0.010 (0.935)^**^ | 0.133 (0.363)^*^ | -0.027 (0.821)^**^ | -0.090 (0.515)^*^ |
| **CCR5+ (%)** | 0.059 (0.631)^**^ | -0.005 (0.970)^*^ | 0.156 (0.185)^**^ | -0.050 (0.720)^*^ |
| **CD38+ (%)** | 0.088 (0.477)^*^ | **0.285 (0.047)**^*^ | 0.154 (0.189)^*^ | 0.196 (0.156)^*^ |
| **CD38+ MFI** | 0.074 (0.549)^**^ | 0.046 (0.751)^*^ | 0.154 (0.189)^**^ | **0.325 (0.017)**^*^ |
| **CD40+ (%)** | -0.176 (0.151)^*^ | 0.181 (0.214)^*^ | -0.194 (0.098)^*^ | -0.015 (0.915)^*^ |
| **CD40+ MFI** | -0.143 (0.244)^*^ | 0.116 (0.429)^*^ | -0.209 (0.074)^*^ | -0.103 (0.460)^*^ |
| **CD163+ (%)** | 0.074 (0.549)^*^ | 0.172 (0.238)^*^ | 0.003 (0.982)^*^ | 0.110 (0.426)^*^ |
| **CD163+ MFI** | -0.001 (0.999)^*^ | 0.135 (0.353)^*^ | -0.115 (0.331)^*^ | -0.050 (0.718)^*^ |
| **CX3CR1+ (%)** | -0.032 (0.797)^*^ | -0.011 (0.940)^*^ | 0.077 (0.512)^*^ | -0.005 (0.969)^*^ |
| **CX3CR1+ MFI** | -0.022 (0.859)^*^ | -0.044 (0.766)^*^ | 0.225 (0.054)^*^ | 0.190 (0.169)^*^ |
| **HLA-DR+ (%)** | 0.150 (0.223)^*^ | 0.234 (0.105)^*^ | 0.100 (0.399)^*^ | 0.027 (0.846)^*^ |
| **HLA-DR+ MFI** | 0.009 (0.942)^*^ | 0.032 (0.829)^*^ | 0.172 (0.143)^*^ | 0.195 (0.159)^*^ |
| **CD11b+ (%)** | 0.113 (0.358)^*^ | **0.311 (0.030)**^*^ | 0.099 (0.403)^*^ | 0.226 (0.100)^*^ |
| **CD11b+ MFI** | 0.203 (0.096)^*^ | **0.360 (0.011)**^*^ | 0.087 (0.464)^*^ | 0.186 (0.179)^*^ |

Notes: BMI= body mass index; CCR2= C-C Motif Chemokine Receptor 2; CCR5= C-C chemokine receptor type 5; CD= cluster of differentiation; CX3CR1= C-X3-C Motif Chemokine Receptor 1;HLA-DR= DR isotype of human leukocyte antigen; FAI= fat attenuation index; LAD= Left Anterior Descending artery; LDL-c= low density lipoprotein cholesterol; MFI=mean fluorescent intensity; RCA=Right Coronary Artery

* Pearson’s Correlation Coefficient. ** Spearman’s Rank Correlation Coefficient

\# partial correlation coefficient after controlling for age, sex, race, LDL-c, lipid lowering medication use and BMI and p value

**Supplementary Table 4: Effect of HIV serostatus on unadjusted associations between monocyte subsets and associated cell surface markers and fat attenuation index (n=74)**

**Supplementary Table 4a: Total Classical Monocytes (CD16dim intermediate/inflammatory and CD16neg)**

|  | **RCA Mean FAI**  Marker x Serostatus Interaction | | **LAD Mean FAI**  Marker x Serostatus Interaction | |
| --- | --- | --- | --- | --- |
|  | Coefficient (95% CI) | p-value | Coefficient (95% CI) | p-value |
| **Total** | -0.015 (-0.162, 0.131) | 0.837 | 0.004 (-0.118, 0.126) | 0.946 |
| **CCR2+ (%)** | **0.251 (0.015, 0.488)** | **0.038** | **0.229 (0.019, 0.439)** | **0.033** |
| **^¥^CCR2+ MFI × 10^-3^** | **0.319 (0.005, 0.634)** | **0.047** | **0.279 (0.094, 0.464)** | **0.004** |
| **CCR5+ (%)** | -0.036 (-0.221, 0.150) | 0.701 | 0.011 (-0.150, 0.173) | 0.890 |
| **CD38+ (%)** | 0.122 (-0.048, 0.293) | 0.157 | 0.066 (-0.086, 0.218) | 0.390 |
| **^¥^CD38+ MFI × 10^-2^** | 0.231 (0.116, 0.578) | 0.188 | 0.161 (-0.022, 0.344) | 0.084 |
| **CD40+ (%)** | 0.020 (-0.056, 0.096) | 0.607 | 0.022 (-0.044, 0.088) | 0.514 |
| **^¥^CD40+ MFI × 10^-1^** | -0.027 (-0.069, 0.014) | 0.196 | -0.003 (-0.036, 0.029) | 0.832 |
| **CD163+ (%)** | -0.001 (-0.031, 0.029) | 0.966 | -0.009 (-0.035, 0.017) | 0.493 |
| **^¥^CD163+ MFI × 10^-2^** | 0.043 (-0.038, 0.124) | 0.292 | -0.001 (-0.070, 0.068) | 0.971 |
| **CX3CR1+ (%)** | -0.032 (-0.079, 0.015) | 0.184 | -0.010 (-0.050, 0.030) | 0.615 |
| **^¥^CX3CR1+ MFI × 10^-3^** | -0.138 (-1.878, 1.602) | 0.875 | -0.577 (-1.855, 0.702 | 0.372 |
| **HLA-DR+ (%)** | 0.037 (-0.063, 0.136) | 0.466 | **0.090 (0.006, 0.175)** | **0.037** |
| **^¥^HLA-DR+ MFI × 10^-2^** | 0.035 (-0.028, 0.099) | 0.272 | **0.056 (0.002, 0.110)** | **0.041** |
| **CD11b+ (%)** | -3.262 (-16.979, 10.454) | 0.636 | -6.027 (-13.547, 1.494) | 0.114 |
| **^¥^CD11b+ MFI × 10^-3^** | -0.141 (-0.442, 0.160) | 0.353 | 0.150 (-0.022, 0.322) | 0.085 |

Notes: CCR2= C-C Motif Chemokine Receptor 2; CCR5= C-C chemokine receptor type 5; CD= cluster of differentiation; CX3CR1= C-X3-C Motif Chemokine Receptor 1; HLA-DR= DR isotype of human leukocyte antigen ; FAI= fat attenuation index; LAD= Left Anterior Descending artery; LDL-c= low density lipoprotein cholesterol; MFI=Mean fluorescent intensity; RCA=Right Coronary Artery

¥ Rescaled

**Supplementary Table 4b: CD16dim Monocytes**

|  | **RCA Mean FAI**  Marker x Serostatus Interaction | | **LAD Mean FAI**  Marker x Serostatus Interaction | |
| --- | --- | --- | --- | --- |
|  | Coefficient (95% CI) | *p*-value | Coefficient (95% CI) | *p*-value |
| **Total** | 0.055 (-0.004, 0.113) | 0.066 | 0.029 (-0.021, 0.078) | 0.250 |
| **CCR2+ (%)** | 0.275 (0.066, 0.485) | **0.011** | **0.211 (0.020, 0.401)** | **0.031** |
| **^¥^CCR2+ MFI × 10^-3^** | 0.345 (0.029, 0.661) | **0.033** | **0.322 (0.113, 0.532)** | **0.003** |
| **CCR5+ (%)** | 0.060 (-0.082, 0.203) | 0.402 | 0.048 (-0.076, 0.172) | 0.442 |
| **CD38+ (%)** | 0.179 (-0.014, 0.372) | 0.068 | 0.033 (-0.134, 0.200) | 0.697 |
| **^¥^CD38+ MFI × 10^-2^** | 0.215 (-0.138, 0.569) | 0.228 | 0.175 (-0.017, 0.367) | 0.073 |
| **CD40+ (%)** | -0.039 (-0.094, 0.016) | 0.157 | -0.018 (-0.066, 0.031) | 0.471 |
| **^¥^CD40+ MFI × 10^-1^** | -0.019 (-0.046, 0.008) | 0.167 | -0.005 (-0.028, 0.018) | 0.663 |
| **CD163+ (%)** | -0.007 (-0.051, 0.037) | 0.755 | 0.006 (-0.033, 0.045) | 0.760 |
| **^¥^CD163+ MFI × 10^-2^** | 0.025 (-0.047, 0.096) | 0.492 | 0.001 (-0.059, 0.062) | 0.961 |
| **CX3CR1+ (%)** | **-0.059 (-0.102, -0.016)** | **0.008** | -0.023 (-0.062, 0.015) | 0.229 |
| **^¥^CX3CR1+ MFI × 10^-3^** | -0.587 (-2.054, 0.879) | 0.427 | -0.586 (-1.625, 0.452) | 0.264 |
| **HLA-DR+ (%)** | 0.047 (-0.048, 0.143) | 0.327 | 0.082 (-0.001, 0.165) | 0.051 |
| **^¥^HLA-DR+ MFI × 10^-2^** | 0.031 (-0.031, 0.093) | 0.322 | **0.054 (0.001, 0.106)** | **0.044** |
| **CD11b+ (%)** | **2.867 (0.069, 5.666)** | **0.045** | 0.504 (-1.836, 2.844) | 0.669 |
| **^¥^CD11b+ MFI × 10^-3^** | -0.111 (0.427, 0.205) | 0.484 | 0.177 (-0.025, 0.379) | 0.085 |

Notes: CCR2= C-C Motif Chemokine Receptor 2; CCR5= C-C chemokine receptor type 5; CD= cluster of differentiation; CX3CR1= C-X3-C Motif Chemokine Receptor 1; HLA-DR= DR isotype of human leukocyte antigen; FAI= fat attenuation index; LAD= Left Anterior Descending artery; LDL-c= low density lipoprotein cholesterol; MFI=Mean fluorescent intensity; RCA=Right Coronary Artery

¥ Rescaled

**Supplementary Table 4c:** **CD16neg Monocytes**

|  | **RCA Mean FAI**  Marker x Serostatus Interaction | | **LAD Mean FAI**  Marker x Serostatus Interaction | |
| --- | --- | --- | --- | --- |
|  | Coefficient (95% CI) | *p*-value | Coefficient (95% CI) | *p*-value |
| **Total** | -0.062 (-0.141, 0.016) | 0.116 | -0.036 (-0.102, 0.031) | 0.287 |
| **CCR2+ (%)** | 0.180 (-0.110, 0.469) | 0.219 | **0.273 (0.033, 0.513)** | **0.026** |
| **^¥^CCR2+ MFI × 10^-3^** | 0.250 (-0.025, 0.524) | 0.074 | **0.222 (0.067, 0.377)** | **0.006** |
| **CCR5+ (%)** | 0.913 (-0.435, 2.260) | 0.181 | 0.535 (-0.096, 1.166) | 0.095 |
| **CD38+ (%)** | 0.073 (-0.078, 0.224) | 0.336 | 0.057 (-0.071, 0.184) | 0.377 |
| **^¥^CD38+ MFI × 10^-2^** | 0.170 (-0.174, 0.514 | 0.326 | 0.144 (-0.003, 0.292) | 0.055 |
| **CD40+ (%)** | -0.009 (-0.062, 0.044) | 0.741 | -0.003 (-0.050, 0.043) | 0.890 |
| **^¥^CD40+ MFI × 10^-1^** | -0.061 (-0.136, 0.014) | 0.108 | -0.019 (-0.076, 0.038) | 0.506 |
| **CD163+ (%)** | -0.022 (-0.056, 0.012) | 0.195 | -0.025 (-0.054, 0.004) | 0.092 |
| **^¥^CD163+ MFI × 10^-2^** | -0.009 (-0.103, 0.084) | 0.845 | -0.022 (-0.101, 0.057) | 0.575 |
| **CX3CR1+ (%)** | **-0.031 (-0.057, -0.004)** | **0.025** | -0.017 (-0.041, 0.006) | 0.147 |
| **^¥^CX3CR1+ MFI × 10^-3^** | -0.752 (-2.504, 1.000) | 0.395 | -0.646 (-2.002, 0.710) | 0.345 |
| **HLA-DR+ (%)** | 0.042 (-0.056, 0.140) | 0.394 | **0.120 (0.040, 0.199)** | **0.004** |
| **^¥^HLA-DR+ MFI × 10^-2^** | 0.037 (-0.028, 0.101) | 0.262 | **0.060 (0.005, 0.115)** | **0.033** |
| **CD11b+ (%)** | -0.869 (-2.789, 1.052) | 0.369 | -0.602 (-2.306, 1.102) | 0.484 |
| **^¥^CD11b+ MFI × 10^-3^** | -0.097 (-0.391, 0.197) | 0.511 | 0.131 (-0.027, 0.288) | 0.103 |

Notes: CCR2= C-C Motif Chemokine Receptor 2; CCR5= C-C chemokine receptor type 5; CD= cluster of differentiation; CX3CR1= C-X3-C Motif Chemokine Receptor 1; HLA-DR= DR isotype of human leukocyte antigen; FAI= fat attenuation index; LAD= Left Anterior Descending artery; LDL-c= low density lipoprotein cholesterol; MFI=Mean fluorescent intensity; RCA=Right Coronary Artery

¥ Rescaled

**Supplementary Table 4d: Intermediate Monocytes**

|  | **RCA Mean FAI**  Marker x Serostatus | | **LAD Mean FAI**  Marker x Serostatus Interaction | |
| --- | --- | --- | --- | --- |
|  | Coefficient (95% CI) | *p*-value | Coefficient (95% CI) | *p*-value |
| **Total** | -0.258 (-1.001, 0.485) | 0.490 | 0.341 (-0.148, 0.831) | 0.168 |
| **CCR2+ (%)** | 0.020 (-0.039, 0.080) | 0.493 | 0.035 (-0.014, 0.085) | 0.155 |
| **^¥^CCR2+ MFI × 10^-3^** | 1.054 (-1.031, 3.139) | 0.316 | **1.812 (0.014, 3.611)** | **0.048** |
| **CCR5+ (%)** | 0.004 (-0.096, 0.104) | 0.937 | 0.016 (-0.055, 0.087) | 0.658 |
| **CD38+ (%)** | 0.030 (-0.012, 0.073) | 0.158 | 0.006 (-0.029, 0.041) | 0.722 |
| **^¥^CD38+ MFI × 10^-2^** | 0.474 (-0.363, 1.311) | 0.262 | -0.050 (-0.764, 0.664) | 0.889 |
| **CD40+ (%)** | -0.030 (-0.095, 0.035) | 0.355 | -0.012 (-0.066, 0.041) | 0.645 |
| **^¥^CD40+ MFI × 10^-1^** | -0.014 (-0.034, 0.007) | 0.187 | -0.001 (-0.019, 0.016) | 0.879 |
| **CD163+ (%)** | 0.009 (-0.031, 0.050) | 0.648 | -0.008 (-0.043, 0.026) | 0.629 |
| **^¥^CD163+ MFI × 10^-2^** | 0.051 (-0.028, 0.130) | 0.204 | 0.011 (-0.058, 0.080) | 0.749 |
| **CX3CR1+ (%)** | -0.034 (-0.147, 0.079) | 0.552 | 0.019 (-0.081, 0.119) | 0.705 |
| **^¥^CX3CR1+ MFI × 10^-3^** | 0.043 (-0.464, 0.378) | 0.840 | -0.288 (-0.582, 0.126) | 0.202 |
| **HLA-DR+ (%)** | -0.192 (-0.470, 0.086) | 0.173 | -0.148 (-0.394, 0.098) | 0.234 |
| **^¥^HLA-DR+ MFI × 10^-2^** | -0.008 (-0.039, 0.023) | 0.607 | -0.001 (-0.027, 0.026) | 0.982 |
| **CD11b+ (%)** | 0.042 (-0.214, 0.298) | 0.744 | -0.112 (-0.307, 0.083) | 0.255 |
| **^¥^CD11b+ MFI × 10^-3^** | -0.079 (-0.481, 0.323) | 0.697 | -0.146 (-0.498, 0.205) | 0.409 |

Notes: CCR2= C-C Motif Chemokine Receptor 2; CCR5= C-C chemokine receptor type 5; CD= cluster of differentiation; CX3CR1= C-X3-C Motif Chemokine Receptor 1; HLA-DR= DR isotype of human leukocyte antigen; FAI= fat attenuation index; LAD= Left Anterior Descending artery; LDL-c= low density lipoprotein cholesterol; MFI=mean fluorescent intensity; RCA=Right Coronary Artery

¥ Rescaled

**Supplementary Table 4e: Non-Classical Monocytes**

|  | **RCA Mean FAI**  Marker x Serostatus Interaction | | **LAD Mean FAI**  Marker x Serostatus Interaction | |
| --- | --- | --- | --- | --- |
|  | Coefficient (95% CI) | *p*-value | Coefficient (95% CI) | *p*-value |
| **Total** | 0.025 (-0.137, 0.187) | 0.761 | -0.034 (-0.173, 0.104) | 0.623 |
| **CCR2+ (%)** | 0.230 (-0.051, 0.512) | 0.107 | 0.179 (-0.067, 0.425) | 0.151 |
| **^¥^CCR2+ MFI × 10^-3^** | -0.087 (-2.912, 2.737) | 0.951 | 2.328 (-0.039, 4.696) | 0.054 |
| **CCR5+ (%)** | -0.006 (-0.122, 0.110) | 0.915 | -0.003 (-0.104, 0.099) | 0.961 |
| **CD38+ (%)** | 0.070 (-0.011, 0.150) | 0.088 | -0.033 (-0.102, 0.036) | 0.345 |
| **^¥^CD38+ MFI × 10^-2^** | 0.316 (-0.332, 0.963) | 0.334 | 0.333 (-0.167, 0.833) | 0.188 |
| **CD40+ (%)** | -0.014 (-0.057, 0.029) | 0.517 | 0.002 (-0.036, 0.040) | 0.906 |
| **^¥^CD40+ MFI × 10^-1^** | -0.018 (-0.046, 0.011) | 0.215 | 0.001 (-0.022, 0.023) | 0.931 |
| **CD163+ (%)** | 0.001 (-0.058, 0.059) | 0.984 | 0.003 (-0.047, 0.053) | 0.904 |
| **^¥^CD163+ MFI × 10^-2^** | 0.035 (-0.107, 0.176) | 0.627 | -0.015 (-0.129, 0.100) | 0.798 |
| **CX3CR1+ (%)** | -0.023 (-0.111, 0.065) | 0.601 | -0.027 (-0.104, 0.050) | 0.481 |
| **^¥^CX3CR1+ MFI × 10^-3^** | 0.097 (-0.048, 0.242) | 0.188 | -0.002 (-0.130, 0.126) | 0.972 |
| **HLA-DR+ (%)** | -0.058 (-0.132, 0.015) | 0.119 | -0.027 (-0.092, 0.039) | 0.418 |
| **^¥^HLA-DR+ MFI × 10^-2^** | -0.004 (-0.075, 0.068) | 0.922 | -0.005 (-0.067, 0.056) | 0.859 |
| **CD11b+ (%)** | -0.013 (-0.059, 0.033) | 0.581 | -0.010 (-0.050, 0.030) | 0.624 |
| **^¥^CD11b+ MFI × 10^-3^** | -0.757 (-2.071, 0.556) | 0.254 | -0.993 (-2.152, 0.166) | 0.092 |

Notes: CCR2= C-C Motif Chemokine Receptor 2; CCR5= C-C chemokine receptor type 5; CD= cluster of differentiation; CX3CR1= C-X3-C Motif Chemokine Receptor 1; HLA-DR= DR isotype of human leukocyte antigen; FAI= fat attenuation index; LAD= Left Anterior Descending artery; LDL-c= low density lipoprotein cholesterol; MFI=mean fluorescent intensity; RCA=Right Coronary Artery;

¥ Rescaled

**Supplementary Table 5: Effect of HIV serostatus on adjusted associations between monocyte subsets and associated cell surface markers and fat attenuation index (n=74)**

**Supplementary Table 5a: Total Classical Monocytes (CD16dim and CD16neg)**

|  | **RCA Mean FAI**  **Marker x Serostatus Interaction** | | **LAD Mean FAI**  **Marker x Serostatus Interaction** | |
| --- | --- | --- | --- | --- |
|  | **Coefficient (95% CI)** | ***p*-value** | **Coefficient (95% CI)** | ***p*-value** |
| **Total** | -0.046 (-0.188, 0.095) | 0.513 | 0.005 (-0.116, 0.126) | 0.933 |
| **CCR2+ (%)** | 0.211 (-0.025, 0.447) | 0.078 | 0.205 (-0.014, 0.424) | 0.066 |
| **^¥^CCR2+ MFI × 10^-3^** | 0.369 (0.096, 0.642) | **0.009** | 0.237 (0.061, 0.414) | **0.009** |
| **CCR5+ (%)** | 0.281 (-0.104, 0.666) | 0.149 | 0.251 (-0.076, 0.578) | 0.129 |
| **CD38+ (%)** | 0.188 (-0.038, 0.273) | 0.134 | 0.055 (-0.092, 0.202) | 0.455 |
| **^¥^CD38+ MFI × 10^-2^** | 0.268 (-0.035, 0.571) | 0.081 | 0.150 (-0.030, 0.329) | 0.100 |
| **CD40+ (%)** | -0.012 (-0.082, 0.059) | 0.740 | -0.004 (-0.068, 0.061) | 0.913 |
| **^¥^CD40+ MFI × 10^-1^** | -0.053 (-0.092, -0.015) | **0.007** | -0.004 (-0.037, 0.028) | 0.782 |
| **CD163+ (%)** | -0.001 (-0.028, 0.027) | 0.988 | -0.005 (-0.031, 0.020) | 0.673 |
| **^¥^CD163+ MFI × 10^-2^** | 0.060 (-0.019, 0.138) | 0.134 | -0.004 (-0.077, 0.068) | 0.902 |
| **CX3CR1+ (%)** | -0.062 (-0.116, -0.008) | **0.025** | 0.006 (-0.044, 0.056) | 0.819 |
| **^¥^CX3CR1+ MFI × 10^-3^** | -0.119 (-1.705, 1.467) | 0.880 | -0.525 (-1.726, 0.676) | 0.384 |
| **HLA-DR+ (%)** | 0.058 (-0.050, 0.166) | 0.287 | 0.102 (0.008, 0.196) | **0.034** |
| **^¥^HLA-DR+ MFI × 10^-2^** | 0.065 (0.006, 0.123) | **0.032** | 0.065 (0.011, 0.119) | **0.020** |
| **CD11b+ (%)** | 0.767 (-12.471, 14.006) | 0.908 | -1.044 (-8.761, 6.673) | 0.787 |
| **^¥^CD11b+ MFI × 10^-3^** | -0.332 (-0.630, -0.034) | **0.030** | 0.137 (-0.038, 0.311) | 0.122 |

Notes: BMI= body mass index; CCR2= C-C Motif Chemokine Receptor 2; CCR5= C-C chemokine receptor type 5; CD= cluster of differentiation; CX3CR1= C-X3-C Motif Chemokine Receptor 1; HLA-DR= DR isotype of human leukocyte antigen ; FAI= fat attenuation index; LAD= Left Anterior Descending artery; LDL-c= low density lipoprotein cholesterol; MFI=Mean fluorescent intensity; RCA=Right Coronary Artery

¥ Rescaled

\# Regression coefficient after adjusting for age, sex, race, LDL-c, lipid lowering medication use and BMI

**Supplementary Table 5b: CD16dim Monocytes**

|  | **RCA Mean FAI**  **Marker x Serostatus Interaction** | | **LAD Mean FAI**  **Marker x Serostatus Interaction** | |
| --- | --- | --- | --- | --- |
|  | **Coefficient (95% CI)** | ***p*-value** | **Coefficient (95% CI)** | ***p*-value** |
| **Total** | 0.008 (-0.052, 0.069) | 0.782 | 0.012 (-0.039, 0.062) | 0.638 |
| **CCR2+ (%)** | 0.202 (-0.013, 0.417) | 0.065 | 0.142 (-0.059, 0.344) | 0.162 |
| **^¥^CCR2+ MFI × 10^-3^** | 0.362 (0.083, 0.641) | **0.012** | 0.258 (0.056, 0.459) | **0.013** |
| **CCR5+ (%)** | 0.356 (0.021, 0.690) | **0.038** | 0.224 (-0.058, 0.506) | 0.117 |
| **CD38+ (%)** | 0.193 (0.008, 0.378) | **0.041** | 0.035 (-0.134, 0.204) | 0.677 |
| **^¥^CD38+ MFI × 10^-2^** | 0.259 (-0.065, 0.584) | 0.114 | 0.154 (-0.028, 0.336) | 0.095 |
| **CD40+ (%)** | -0.072 (-0.123, -0.021) | **0.006** | -0.026 (-0.076, 0.024) | 0.295 |
| **^¥^CD40+ MFI × 10^-1^** | -0.033 (-0.060, -0.007) | **0.014** | -0.003 (-0.027, 0.021) | 0.781 |
| **CD163+ (%)** | -0.012 (-0.051, 0.028) | 0.558 | 0.004 (-0.033, 0.041) | 0.834 |
| **^¥^CD163+ MFI × 10^-2^** | 0.058 (-0.010, 0.126) | 0.090 | 0.009 (-0.053, 0.072) | 0.766 |
| **CX3CR1+ (%)** | -0.065 (-0.112, -0.019) | **0.007** | -0.003 (-0.048, 0.042) | 0.895 |
| **^¥^CX3CR1+ MFI × 10^-3^** | -0.216 (-1.544, 1.112) | 0.745 | -0.419 (-1.377, 0.538) | 0.383 |
| **HLA-DR+ (%)** | 0.072 (-0.028, 0.171) | 0.153 | 0.085 (-0.005, 0.175) | 0.065 |
| **^¥^HLA-DR+ MFI × 10^-2^** | 0.056 (-0.001, 0.114) | 0.055 | 0.058 (0.004, 0.111) | **0.034** |
| **CD11b+ (%)** | 2.098 (-0.555, 4.750) | 0.118 | -0.753 (-3.014, 1.508) | 0.507 |
| **^¥^CD11b+ MFI × 10^-3^** | -0.329 (-0.644, -0.014) | **0.041** | 0.162 (-0.047, 0.371) | 0.125 |

Notes: BMI= body mass index; CCR2= C-C Motif Chemokine Receptor 2; CCR5= C-C chemokine receptor type 5; CD= cluster of differentiation; CX3CR1= C-X3-C Motif Chemokine Receptor 1; HLA-DR= DR isotype of human leukocyte antigen; FAI= fat attenuation index; LAD= Left Anterior Descending artery; LDL-c= low density lipoprotein cholesterol; MFI=Mean fluorescent intensity; RCA=Right Coronary Artery

¥ Rescaled

\# Regression coefficient after adjusting for age, sex, race, LDL-c, lipid lowering medication use and BMI

**Supplementary Table 5c: CD16neg Monocytes**

|  | **RCA Mean FAI**  **Marker x Serostatus Interaction** | | **LAD Mean FAI**  **Marker x Serostatus Interaction** | |
| --- | --- | --- | --- | --- |
|  | **Coefficient (95% CI)** | ***p*-value** | **Coefficient (95% CI)** | ***p*-value** |
| **Total** | -0.024 (-0.104, 0.057) | 0.557 | -0.018 (-0.086, 0.051) | 0.604 |
| **CCR2+ (%)** | 0.246 (-0.061, 0.552) | 0.114 | 0.254 (-0.020, 0.528) | 0.069 |
| **^¥^CCR2+ MFI × 10^-3^** | 0.324 (0.083, 0.556) | **0.010** | 0.178 (0.029, 0.328) | **0.020** |
| **CCR5+ (%)** | 1.077 (-0.220, 2.374) | 0.101 | 0.445 (-0.151, 1.042) | 0.140 |
| **CD38+ (%)** | 0.152 (0.007, 0.297) | **0.041** | 0.075 (-0.056, 0.206) | 0.257 |
| **^¥^CD38+ MFI × 10^-2^** | 0.255 (-0.057, 0.568) | 0.107 | 0.119 (-0.020, 0.257) | 0.092 |
| **CD40+ (%)** | 0.09 (-0.044, 0.061) | 0.738 | 0.006 (-0.043, 0.055) | 0.820 |
| **^¥^CD40+ MFI × 10^-1^** | -0.058 (-0.130, 0.013) | 0.108 | 0.005 (-0.050, 0.060) | 0.845 |
| **CD163+ (%)** | -0.015 (-0.046, 0.017) | 0.353 | -0.016 (-0.045, 0.013) | 0.265 |
| **^¥^CD163+ MFI × 10^-2^** | 0.011 (-0.081, 0.102) | 0.817 | -0.009 (-0.090, 0.073) | 0.827 |
| **CX3CR1+ (%)** | -0.037 (-0.063, -0.011) | **0.006** | -0.010 (-0.035, 0.015) | 0.435 |
| **^¥^CX3CR1+ MFI × 10^-3^** | -0.776 (-2.484, 0.931) | 0.365 | -0.554 (-1.892, 0.784) | 0.410 |
| **HLA-DR+ (%)** | 0.069 (-0.035, 0.174) | 0.187 | 0.141 (0.057, 0.225) | **0.001** |
| **^¥^HLA-DR+ MFI × 10^-2^** | 0.081 (0.018, 0.144) | **0.013** | 0.079 (0.021, 0.138) | **0.009** |
| **CD11b+ (%)** | -0.985 (-6.032, 4.061) | 0.696 | 2.070 (-2.611, 6.752) | 0.379 |
| **^¥^CD11b+ MFI × 10^-3^** | -0.233 (-0.536, 0.070) | 0.129 | 0.123 (-0.034, 0.280) | 0.123 |

Notes: BMI= body mass index; CCR2= C-C Motif Chemokine Receptor 2; CCR5= C-C chemokine receptor type 5; CD= cluster of differentiation; CX3CR1= C-X3-C Motif Chemokine Receptor 1; HLA-DR= DR isotype of human leukocyte antigen; FAI= fat attenuation index; LAD= Left Anterior Descending artery; LDL-c= low density lipoprotein cholesterol; MFI=Mean fluorescent intensity; RCA=Right Coronary Artery

¥ Rescaled

\# Regression coefficient after adjusting for age, sex, race, LDL-c, lipid lowering medication use and BMI

**Supplementary Table 5d: Intermediate Monocytes**

|  | **RCA Mean FAI**  **Marker x Serostatus** | | **LAD Mean FAI**  **Marker x Serostatus Interaction** | |
| --- | --- | --- | --- | --- |
|  | **Coefficient (95% CI)** | ***p*-value** | **Coefficient (95% CI)** | ***p*-value** |
| **Total** | -0.073 (-0.743, 0.597) | 0.827 | 0.323 (-0.136, 0.782) | 0.164 |
| **CCR2+ (%)** | 0.001 (-0.058, 0.059) | 0.999 | 0.032 (-0.017, 0.082) | 0.195 |
| **^¥^CCR2+ MFI × 10^-3^** | 1.406 (-0.469, 3.282) | 0.138 | 1.553 (-0.200, 3.305) | 0.081 |
| **CCR5+ (%)** | -0.037 (-0.153, 0.079) | 0.526 | 0.020 (-0.074, 0.114) | 0.671 |
| **CD38+ (%)** | 0.019 (-0.022, 0.061) | 0.358 | 0.006 (-0.029, 0.041) | 0.737 |
| **^¥^CD38+ MFI × 10^-2^** | 0.496 (-0.404, 1.395) | 0.273 | 0.208 (-0.591, 1.007) | 0.604 |
| **CD40+ (%)** | -0.096 (-0.161, -0.032) | **0.004** | -0.024 (-0.080, 0.031) | 0.382 |
| **^¥^CD40+ MFI × 10^-1^** | -0.032 (-0.050, -0.013) | **0.002** | -0.003 (-0.022, 0.016) | 0.732 |
| **CD163+ (%)** | 0.007 (-0.032, 0.045) | 0.726 | -0.003 (-0.037, 0.032) | 0.883 |
| **^¥^CD163+ MFI × 10^-2^** | 0.103 (0.022, 0.183) | **0.014** | 0.034 (-0.043, 0.111) | 0.377 |
| **CX3CR1+ (%)** | -0.080 (-0.312, 0.151) | 0.489 | 0.123 (-0.093, 0.339) | 0.259 |
| **^¥^CX3CR1+ MFI × 10^-3^** | 0.086 (-0.319, 0.491) | 0.670 | -0.214 (-0.568, 0.140) | 0.230 |
| **HLA-DR+ (%)** | -0.116 (-0.391, 0.159) | 0.400 | -0.008 (-0.265, 0.249) | 0.952 |
| **^¥^HLA-DR+ MFI × 10^-2^** | 0.028 (-0.004, 0.061) | 0.087 | 0.019 (-0.011, 0.049) | 0.215 |
| **CD11b+ (%)** | 0.150 (-0.091, 0.392) | 0.215 | 0.021 (-0.185, 0.227) | 0.841 |
| **^¥^CD11b+ MFI × 10^-3^** | -0.106 (-0.527, 0.315) | 0.614 | 0.013 (-0.377, 0.403) | 0.948 |

Notes: BMI= body mass index; CCR2= C-C Motif Chemokine Receptor 2; CCR5= C-C chemokine receptor type 5; CD= cluster of differentiation; CX3CR1= C-X3-C Motif Chemokine Receptor 1; HLA-DR= DR isotype of human leukocyte antigen; FAI= fat attenuation index; LAD= Left Anterior Descending artery; LDL-c= low density lipoprotein cholesterol; MFI=mean fluorescent intensity; RCA=Right Coronary Artery

¥ Rescaled

\# Regression coefficient after adjusting for age, sex, race, LDL-c, lipid lowering medication use and BMI

**Supplementary Table 5e: Non-Classical Monocytes**

|  | **RCA Mean FAI**  **Marker x Serostatus Interaction** | | **LAD Mean FAI**  **Marker x Serostatus Interaction** | |
| --- | --- | --- | --- | --- |
|  | **Coefficient (95% CI)** | ***p*-value** | **Coefficient (95% CI)** | ***p*-value** |
| **Total** | 0.060 (-0.098, 0.218) | 0.446 | -0.036 (-0.177, 0.106) | 0.612 |
| **CCR2+ (%)** | 0.041 (-0.241, 0.323) | 0.773 | 0.113 (-0.134, 0.360) | 0.362 |
| **^¥^CCR2+ MFI × 10^-3^** | -0.631 (-3.083, 1.821) | 0.607 | 2.053 (-0.157, 4.263) | 0.068 |
| **CCR5+ (%)** | 0.003 (-0.161, 0.167) | 0.972 | 0.072 (-0.076, 0.219) | 0.333 |
| **CD38+ (%)** | 0.041 (-0.030, 0.111) | 0.248 | -0.033 (-0.099, 0.033) | 0.316 |
| **^¥^CD38+ MFI × 10^-2^** | 0.513 (-0.059, 1.086) | 0.078 | 0.412 (-0.097, 0.920) | 0.110 |
| **CD40+ (%)** | -0.048 (-0.092, -0.004) | **0.032** | -0.011 (-0.055, 0.032) | 0.601 |
| **^¥^CD40+ MFI × 10^-1^** | -0.036 (-0.063, -0.010) | **0.008** | -0.003 (-0.027, 0.021) | 0.816 |
| **CD163+ (%)** | 0.009 (-0.049, 0.067) | 0.756 | 0.015 (-0.037, 0.068) | 0.563 |
| **^¥^CD163+ MFI × 10^-2^** | 0.140 (-0.003, 0.284) | 0.055 | 0.046 (-0.078, 0.170) | 0.457 |
| **CX3CR1+ (%)** | -0.003 (-0.202, 0.195) | 0.973 | -0.029 (-0.211, 0.153) | 0.754 |
| **^¥^CX3CR1+ MFI × 10^-3^** | 0.122 (-0.005, 0.250) | 0.060 | -0.048 (-0.169, 0.073) | 0.430 |
| **HLA-DR+ (%)** | -0.033 (-0.107, 0.040) | 0.365 | -0.003 (-0.073, 0.067) | 0.923 |
| **^¥^HLA-DR+ MFI × 10^-2^** | 0.073 (0.001, 0.144) | **0.046** | 0.017 (-0.049, 0.084) | 0.602 |
| **CD11b+ (%)** | -0.008 (-0.053, 0.037) | 0.724 | 0.007 (-0.036, 0.049) | 0.753 |
| **^¥^CD11b+ MFI × 10^-3^** | -0.668 (-1.856, 0.520) | 0.263 | -0.611 (-1.773, 0.552) | 0.296 |

Notes: BMI= body mass index; CCR2= C-C Motif Chemokine Receptor 2; CCR5= C-C chemokine receptor type 5; CD= cluster of differentiation; CX3CR1= C-X3-C Motif Chemokine Receptor 1; HLA-DR= DR isotype of human leukocyte antigen; FAI= fat attenuation index; LAD= Left Anterior Descending artery; LDL-c= low density lipoprotein cholesterol; MFI=mean fluorescent intensity; RCA=Right Coronary Artery;

¥ Rescaled

\# Regression coefficient after adjusting for age, sex, race, LDL-c, lipid lowering medication use and BMI

**Supplementary Table 6: Unadjusted Correlations Between Soluble Marker and Monocyte Subset and Chemokine Receptor Expression (n=79)**

**Supplementary Table 6a: Classical Monocytes**

|  | **All Study Participants (Persons with HIV and without HIV) n=79** | | | | | | | | | |
| --- | --- | --- | --- | --- | --- | --- | --- | --- | --- | --- |
| **Soluble Marker** | **IL-6** | **Hs-CRP** | **TNFR-1** | **TNFR-2** | **CCL2** | **CCL5** | **CX3CL1** | **CXCL10** | **sCD14** | **sCD163** |
|  | ***r**  **(p value)** | ***r**  **(p value)** | ***r**  **(p value)** | ***r**  **(p value)** | ***r**  **(p value)** | ***r**  **(p value)** | ***r**  **(p value)** | ***r**  **(p value)** | ***r**  **(p value)** | ***r**  **(p value)** |
| **Total** | -0.037  (0.754) | -0.110  (0.349) | 0.010  (0.936) | -0.026  (0.823) | -0.053  (0.654) | 0.120  (0.308) | -0.102  (0.385) | -0.207  (0.077) | -0.032  (0.789) | -0.106  (0.369) |
| **CCR2+ (%)** | -0.019  (0.873) | -0.098  (0.404) | -0.034  (0.771) | -0.003  (0.978) | -0.085  (0.469) | 0.153  (0.194) | 0.222  (0.057) | 0.151  (0.198) | -0.181  (0.124) | 0.115  (0.328) |
| **^¥^CCR2+ MFI × 10^-3^** | -0.071  (0.548) | -0.139  (0.238) | -0.047  (0.692) | 0.014  (0.903) | 0.022  (0.853) | -0.033  (0.780) | **0.248**  **(0.034)** | 0.107  (0.363) | **-0.327**  **(0.005)** | 0.005  (0.964) |
| **CCR5+ (%)** | 0.057  (0.628) | 0.047  (0.692) | 0.102  (0.389) | 0.069  (0.556) | 0.066  (0.576) | **-0.235**  **(0.044)** | 0.008  (0.944) | 0.060  (0.614) | **0.272**  **(0.020)** | -0.038  (0.749) |
| **CD38+ (%)** | -0.099  (0.398) | -0.168  (0.153) | -0.061  (0.605) | -0.051  (0.668) | -0.076  (0.521) | -0.108  (0.358) | 0.147  (0.211) | 0.186  (0.113) | 0.141  (0.229) | 0.090  (0.447) |
| **^¥^CD38+ MFI × 10^-2^** | -0.026  (0.828) | -0.081  (0.491) | -0.092  (0.435) | -0. 019  (0.870) | -0.014  (0.908) | -0.206  (0.079) | 0.116  (0.324) | 0.217  (0.064) | 0.027  (0.817) | 0.107  (0.364) |
| **CD40+ (%)** | 0.057  (0.628) | -0.038  (0.748) | 0.008  (0.944) | -0.021  (0.857) | 0.014  (0.907) | 0.188  (0.109) | 0.075  (0.525) | 0.153  (0.193) | -0.015  (0.900) | 0.121  (0.306) |
| **^¥^CD40+ MFI × 10^-1^** | 0.057  (0.630) | -0.003  (0.978) | -0.025  (0.834) | -0.038  (0.750) | -0.043  (0.718) | 0.105  (0.374) | -0.206  (0.079) | 0.011  (0.927) | 0.056  (0.637) | -0.001  (0.999) |
| **CD163+ (%)** | -0.127  (0.282) | **-0.250**  **(0.032)** | 0.092  (0.435) | -0.026  (0.825) | **-0.229**  **(0.050)** | 0.021  (0.859) | 0.049  (0.677) | 0.006  (0.962) | 0.071  (0.547) | -0.178  (0.130) |
| **^¥^CD163+ MFI × 10^-2^** | -0.077  (0.513) | **-0.238**  **(0.041)** | -0.079  (0.502) | -0.053  (0.651) | -0.150  (0.203) | -0.078  (0.511) | 0.195  (0.097) | 0.155  (0.186) | **-0.258**  **(0.027)** | -0.002  (0.986) |
| **CX3CR1+ (%)** | -0.005  (0.970) | -0.203  (0.083) | 0.115  (0.330) | 0.013  (0.909) | -0.111  (0.348) | 0.133  (0.257) | 0.226  (0.053) | 0.032  (0.784) | 0.020  (0.867) | -0.010  (0.932) |
| **^¥^CX3CR1+ MFI × 10^-3^** | 0.036  (0.763) | -0.101  (0.391) | 0.007  (0.954) | 0.014  (0.908) | -0.120  (0.307) | 0.015  (0.902) | 0.009  (0.941) | 0.092  (0.433) | -0.081  (0.493) | 0.088  (0.457) |
| **HLA-DR+ (%)** | 0.018  (0.882) | 0.044  (0.707) | -0.024  (0.841) | -0.023  (0.844) | 0.077  (0.515) | -0.008  (0.948) | 0.210  (0.073) | 0.203  (0.084) | 0.078  (0.509) | 0.068  (0.566) |
| **^¥^HLA-DR+ MFI × 10^-2^** | -0.057  (0.630) | -0.029  (0.804) | -0.078  (0.511) | -0.052  (0.657) | 0.052  (0.657) | -0.116  (0.325) | **0.304**  **(0.009)** | 0.210  (0.072) | **-0.283**  **(0.015)** | 0.071  (0.547) |
| **CD11b+ (%)** | 0.032  (0.788) | 0.132  (0.261) | 0.184  (0.117) | 0.216  (0.065) | -0.102  (0.387) | -0.012  (0.918) | -0.132  (0.262) | 0.074  (0.532) | 0.204  (0.082) | 0.113  (0.337) |
| **^¥^CD11b+ MFI × 10^-3^** | -0.045  (0.705) | 0.057  (0.629) | -0.087  (0.460) | -0.105  (0.373) | -0.083  (0.481) | 0.088  (0.455) | -0.226  (0.054) | -0.111  (0.344) | **0.234**  **(0.045)** | -0.023  (0.844) |

Notes: CCR2= C-C Motif Chemokine Receptor 2; CCR5= C-C chemokine receptor type 5; CD= cluster of differentiation; CX3CR1= C-X3-C Motif Chemokine Receptor 1; HLA-DR= DR isotype of human leukocyte antigen; hs-CRP, high-sensitivity C-reactive protein; IL-6, interleukin-6; IP-10, IFN-γ-induced protein 10; MFI= mean fluorescent intensity; TNFR, tumor necrosis factor receptor

Bolded values represent statistically significant results

¥ Rescaled

*Spearman Rank correlation coefficient

**Supplementary Table 6b: CD16dim Monocytes**

|  | **All Study Participants (Persons with HIV and without HIV) n=79** | | | | | | | | | |
| --- | --- | --- | --- | --- | --- | --- | --- | --- | --- | --- |
| **Soluble Marker** | **IL-6** | **Hs-CRP** | **TNFR-1** | **TNFR-2** | **CCL2** | **CCL5** | **CX3CL1** | **CXCL10** | **sCD14** | **sCD163** |
|  | ***r**  **(p value)** | ***r**  **(p value)** | ***r**  **(p value)** | ***r**  **(p value)** | ***r**  **(p value)** | ***r**  **(p value)** | ***r**  **(p value)** | ***r**  **(p value)** | ***r**  **(p value)** | ***r**  **(p value)** |
| **Total** | 0.018  (0.880) | -0.212  (0.070) | 0.009  (0.940) | 0.046  (0.696) | -0.140  (0.233) | 0.101  (0.391) | -0.013  (0.912) | 0.067  (0.568) | **0.259**  **(0.026)** | -0.120  (0.306) |
| **CCR2+ (%)** | 0.001  (0.999) | -0.084  (0.475) | -0.051  (0.665) | -0.058  (0.622) | 0.011  (0.925) | 0.152  (0.195) | **0.280**  **(0.016)** | 0.148  (0.208) | -0.175  (0.136) | 0.084  (0.478) |
| **^¥^CCR2+ MFI × 10^-3^** | -0.032  (0.786) | -0.086  (0.465) | -0.090  (0.447) | -0.050  (0.675) | 0.076  (0.520) | -0.068  (0.566) | **0.323**  **(0.005)** | 0.138  (0.242) | **-0.393**  **(0.001)** | 0.004  (0.975) |
| **CCR5+ (%)** | -0.100  (0.396) | -0.113  (0.336) | 0.071  (0.545) | 0.095  (0.418) | -0.059  (0.615) | -0.199  (0.089) | 0.020  (0.867) | 0.123  (0.298) | 0.210  (0.072) | -0.111  (0.347) |
| **CD38+ (%)** | -0.136  (0.249) | -0.174  (0.137) | -0.093  (0.428) | -0.089  (0.451) | 0.004  (0.975) | 0.012  (0.922) | **0.252**  **(0.031)** | **0.252**  **(0.031)** | 0.121  (0.306) | 0.075  (0.524) |
| **^¥^CD38+ MFI × 10^-2^** | 0.023  (0.843) | -0.025  (0.835) | -0.124  (0.291) | -0.061  (0.602) | 0.059  (0.615) | -0.186  (0.112) | 0.103  (0.381) | 0.158  (0.179) | 0.141  (0.231) | 0.051  (0.664) |
| **CD40+ (%)** | -0.004  (0.973) | 0.027  (0.818) | -0.015  (0.896) | -0.039  (0.740) | 0.178  (0.129) | -0.044  (0.711) | 0.049  (0.679) | **0.245**  **(0.036)** | -0.002  (0.986) | **0.236**  **(0.043)** |
| **^¥^CD40+ MFI × 10^-1^** | 0.021  (0.861) | 0.048  (0.687) | -0.033  (0.779) | -0.081  (0.490) | 0.109  (0.356) | 0.044  (0.710) | -0.168  (0.152) | -0.017  (0.888) | 0.078  (0.510) | 0.046  (0.700) |
| **CD163+ (%)** | -0.191  (0.102) | -0.112  (0.342) | 0.067  (0.568) | 0.010  (0.936) | -0.018  (0.879) | -0.167  (0.156) | 0.161  (0.171) | 0.114  (0.334) | -0.110  (0.349) | 0.064  (0.587) |
| **^¥^CD163+ MFI × 10^-2^** | -0.092  (0.436) | -0.059  (0.617) | 0.002  (0.989) | 0.008  (0.946) | 0.008  (0.945) | -0.187  (0.111) | 0.155  (0.188) | 0.185  (0.115) | **-0.282**  **(0.015)** | 0.063  (0.594) |
| **CX3CR1+ (%)** | -0.090  (0.445) | -0.200  (0.088) | 0.083  (0.479) | -0.020  (0.866) | -0.013  (0.914) | 0.105  (0.372) | 0.122  (0.300) | -0.073  (0.537) | 0.056  (0.638) | 0.046  (0.699) |
| **^¥^CX3CR1+ MFI × 10^-3^** | -0.093  (0.429) | -0.160  (0.174) | -0.074  (0.528) | -0.088  (0.454) | -0.069  (0.557) | 0.038  (0.747) | -0.030  (0.797) | -0.012  (0.918) | -0.134  (0.254) | 0.077  (0.512) |
| **HLA-DR+ (%)** | -0.006  (0.963) | 0.009  (0.936) | -0.044  (0.708) | -0.051  (0.667) | 0.091  (0.440) | 0.034  (0.775) | **0.233**  **(0.046)** | 0.203  (0.082) | -0.034  (0.771) | -0.016  (0.890) |
| **^¥^HLA-DR+ MFI × 10^-2^** | -0.084  (0.478) | -0.049  (0.681) | -0.061  (0.602) | -0.060  (0.609) | 0.069  (0.558) | -0.063  (0.591) | **0.278**  **(0.017)** | 0.194  (0.099) | **-0.260**  **(0.026)** | 0.047  (0.692) |
| **CD11b+ (%)** | -0.030  (0.798) | 0.034  (0.770) | -0.073  (0.539) | 0.025  (0.831) | -0.122  (0.301) | 0.041  (0.731) | -0.016  (0.895) | 0.193  (0.099) | 0.082  (0.487) | 0.091  (0.438) |
| **^¥^CD11b+ MFI × 10^-3^** | -0.010  (0.935) | 0.008  (0.945) | -0.128  (0.277) | -0.144  (0.221) | -0.106  (0.369) | 0.067  (0.571) | -0.223  (0.056) | -0.091  (0.439) | **0.236**  **(0.043)** | -0.073  (0.539) |

Notes: CCR2= C-C Motif Chemokine Receptor 2; CCR5= C-C chemokine receptor type 5; CD= cluster of differentiation; CX3CR1= C-X3-C Motif Chemokine Receptor 1; HLA-DR= DR isotype of human leukocyte antigen; hs-CRP, high-sensitivity C-reactive protein; IL-6, interleukin-6; IP-10, IFN-γ-induced protein 10; MFI= mean fluorescent intensity; TNFR, tumor necrosis factor receptor

Bolded values represent statistically significant results

¥ Rescaled

*Spearman Rank correlation coefficient

**Supplementary Table 6c: CD16neg Monocytes**

|  | **All Study Participants (Persons with HIV and without HIV) n=79** | | | | | | | | | |
| --- | --- | --- | --- | --- | --- | --- | --- | --- | --- | --- |
| **Soluble Marker** | **IL-6** | **Hs-CRP** | **TNFR-1** | **TNFR-2** | **CCL2** | **CCL5** | **CX3CL1** | **CXCL10** | **sCD14** | **sCD163** |
|  | ***r**  **(p value)** | ***r**  **(p value)** | ***r**  **(p value)** | ***r**  **(p value)** | ***r**  **(p value)** | ***r**  **(p value)** | ***r**  **(p value)** | ***r**  **(p value)** | ***r**  **(p value)** | ***r**  **(p value)** |
| **Total** | -0.039  (0.744) | 0.117  (0.320) | -0.013  (0.915) | -0.083  (0.482) | 0.030  (0.799) | 0.008  (0.946) | -0.141  (0.229) | -0.170  (0.147) | -0.220  (0.060) | 0.060  (0.608) |
| **CCR2+ (%)** | 0.048  (0.682) | 0.024  (0.840) | -0.066  (0.579) | -0.050  (0.670) | 0.052  (0.662) | 0.140  (0.233) | **0.303**  **(0.009)** | 0.185  (0.114) | **-0.248**  **(0.034)** | **0.259**  **(0.026)** |
| **^¥^CCR2+ MFI × 10^-3^** | -0.047  (0.688) | -0.087  (0.460) | -0.112  (0.341) | -0.082  (0.485) | 0.068  (0.565) | -0.028  (0.815) | **0.312**  **(0.007)** | 0.122  (0.300) | **-0.409**  **(0.001)** | 0.023  (0.844) |
| **CCR5+ (%)** | 0.102  (0.384) | -0.068  (0.564) | 0.086  (0.468) | 0.094  (0.426) | 0.183  (0.118) | 0.001  (0.997) | 0.090  (0.447) | 0.102  (0.385) | 0.129  (0.271) | -0.034  (0.771) |
| **CD38+ (%)** | -0.059  (0.620) | -0.079  (0.502) | -0.107  (0.365) | -0.087  (0.461) | 0.109  (0.356) | -0.077  (0.513) | **0.309**  **(0.008)** | **0.287**  **(0.013)** | 0.022  (0.855) | 0.170  (0.148) |
| **^¥^CD38+ MFI × 10^-2^** | 0.126  (0.283) | 0.013  (0.910) | -0.075  (0.524) | -0.018  (0.881) | 0.054  (0.645) | **-0.253**  **(0.030)** | 0.152  (0.196) | 0.187  (0.110) | 0.139  (0.239) | 0.056  (0.633) |
| **CD40+ (%)** | 0.144  (0.222) | -0.001  (0.999) | -0.003  (0.983) | 0.013  (0.916) | **0.247**  **(0.034)** | 0.017  (0.889) | 0.224  (0.056) | **0.291**  **(0.012)** | -0.143  (0.225) | 0.181  (0.122) |
| **^¥^CD40+ MFI × 10^-1^** | 0.082  (0.485) | -0.014  (0.906) | -0.206  (0.079) | -0.174  (0.138) | 0.188  (0.108) | 0.073  (0.536) | 0.025  (0.834) | 0.146  (0.214) | -0.227  (0.052) | 0.173  (0.140) |
| **CD163+ (%)** | -0.101  (0.391) | **-0.320**  **(0.006)** | -0.132  (0.262) | -0.156  (0.185) | -0.042  (0.719) | -0.051  (0.664) | 0.149  (0.204) | 0.168  (0.152) | -0.220  (0.060) | 0.037  (0.753) |
| **^¥^CD163+ MFI × 10^-2^** | -0.068  (0.566) | **-0.245**  **(0.035)** | -0.184  (0.118) | -0.148  (0.209) | -0.055  (0.641) | -0.021  (0.857) | 0.135  (0.252) | 0.173  (0.140) | **-0.296**  **(0.011)** | 0.040  (0.735) |
| **CX3CR1+ (%)** | -0.010  (0.936) | **-0.236**  **(0.043)** | 0.100  (0.395) | 0.022  (0.852) | 0.145  (0.217) | 0.062  (0.599) | 0.204  (0.081) | 0.024  (0.838) | -0.119  (0.314) | 0.077  (0.515) |
| **^¥^CX3CR1+ MFI × 10^-3^** | 0.073  (0.535) | -0.147  (0.212) | 0.002  (0.984) | -0.028  (0.813) | 0.218  (0.062) | 0.078  (0.507) | 0.141  (0.230) | 0.075  (0.525) | -0.215  (0.066) | 0.095  (0.421) |
| **HLA-DR+ (%)** | 0.008  (0.948) | 0.060  (0.611) | -0.093  (0.431) | -0.090  (0.446) | 0.143  (0.224) | 0.016  (0.891) | **0.270**  **(0.020)** | **0.232**  **(0.047)** | -0.102  (0.388) | 0.075  (0.525) |
| **^¥^HLA-DR+ MFI × 10^-2^** | -0.097  (0.412) | -0.026  (0.823) | -0.107  (0.365) | -0.081  (0.490) | 0.085  (0.474) | -0.098  (0.407) | **0.308**  **(0.008)** | 0.200  (0.088) | **-0.321**  **(0.006)** | 0.051  (0.668) |
| **CD11b+ (%)** | -0.036  (0.761) | 0.032  (0.784) | 0.099  (0.400) | 0.145  (0.218) | -0.031  (0.794) | -0.143  (0.224) | -0.127  (0.279) | **0.246**  **(0.035)** | 0.062  (0.600) | 0.125  (0.288) |
| **^¥^CD11b+ MFI × 10^-3^** | -0.036  (0.766) | 0.122  (0.300) | -0.068  (0.564) | -0.092  (0.435) | -0.059  (0.619) | 0.049  (0.681) | -0.211  (0.071) | -0.028  (0.811) | 0.214  (0.068) | -0.039  (0.744) |

Notes: CCR2= C-C Motif Chemokine Receptor 2; CCR5= C-C chemokine receptor type 5; CD= cluster of differentiation; CX3CR1= C-X3-C Motif Chemokine Receptor 1; HLA-DR= DR isotype of human leukocyte antigen; hs-CRP, high-sensitivity C-reactive protein; IL-6, interleukin-6; IP-10, IFN-γ-induced protein 10; MFI= mean fluorescent intensity; TNFR, tumor necrosis factor receptor

Bolded values represent statistically significant results

¥ Rescaled

*Spearman Rank correlation coefficient

**Supplementary Table 6d: Intermediate Monocytes**

|  | **All Study Participants (Persons with HIV and without HIV) n=79** | | | | | | | | | |
| --- | --- | --- | --- | --- | --- | --- | --- | --- | --- | --- |
| **Soluble Marker** | **IL-6** | **Hs-CRP** | **TNFR-1** | **TNFR-2** | **CCL2** | **CCL5** | **CX3CL1** | **CXCL10** | **sCD14** | **sCD163** |
|  | ***r**  **(p value)** | ***r**  **(p value)** | ***r**  **(p value)** | ***r**  **(p value)** | ***r**  **(p value)** | ***r**  **(p value)** | ***r**  **(p value)** | ***r**  **(p value)** | ***r**  **(p value)** | ***r**  **(p value)** |
| **Total** | 0.133  (0.257) | 0.026  (0.828) | 0.100  (0.398) | 0.166  (0.157) | 0.067  (0.572) | -0.129  (0.271) | 0.047  (0.688) | 0.164  (0.163) | 0.153  (0.192) | 0.175  (0.136) |
| **CCR2+ (%)** | 0.044  (0.708) | -0.098  (0.406) | -0.011  (0.925) | 0.102  (0.386) | -0.143  (0.223) | -0.013  (0.910) | 0.093  (0.431) | **0.373**  **(0.001)** | 0.076  (0.519) | 0.044  (0.711) |
| **^¥^CCR2+ MFI × 10^-3^** | -0.004  (0.974) | -0.013  (0.910) | -0.033  (0.781) | 0.078  (0.508) | 0.003  (0.979) | -0.047  (0.689) | 0.172  (0.142) | **0.236**  **(0.043)** | **-0.288**  **(0.013)** | 0.009  (0.937) |
| **CCR5+ (%)** | 0.036  (0.759) | -0.016  (0.894) | 0.084  (0.477) | 0.030  (0.801) | -0.012  (0.919) | -0.050  (0.670) | -0.045  (0.702) | 0.065  (0.582) | **0.253**  **(0.030)** | -0.134  (0.254) |
| **CD38+ (%)** | -0.079  (0.504) | -0.127  (0.280) | 0.027  (0.821) | 0.076  (0.521) | -0.138  (0.241) | -0.058  (0.624) | 0.018  (0.881) | **0.294**  **(0.011)** | 0.182  (0.121) | 0.067  (0.571) |
| **^¥^CD38+ MFI × 10^-2^** | -0.056  (0.636) | -0.013  (0.909) | 0.015  (0.900) | 0.117  (0.321) | -0.099  (0.403) | -0.107  (0.363) | -0.084  (0.475) | **0.261**  **(0.025)** | 0.164  (0.162) | 0.071  (0.545) |
| **CD40+ (%)** | 0.064  (0.588) | -0.061  (0.607) | -0.041  (0.728) | 0.018  (0.881) | -0.027  (0.821) | 0.042  (0.725) | -0.027  (0.818) | **0.258**  **(0.027)** | 0.099  (0.403) | 0.207  (0.077) |
| **^¥^CD40+ MFI × 10^-1^** | 0.047  (0.691) | -0.048  (0.685) | -0.094  (0.423) | -0.023  (0.843) | -0.033  (0.780) | 0.007  (0.956) | -0.194  (0.097) | 0.144  (0.220) | 0.061  (0.608) | 0.166  (0.157) |
| **CD163+ (%)** | -0.085  (0.472) | -0.105  (0.371) | 0.023  (0.848) | -0.059  (0.620) | -0.174  (0.139) | 0.024  (0.841) | 0.082  (0.488) | 0.138  (0.240) | 0.105  (0.371) | -0.170  (0.148) |
| **^¥^CD163+ MFI × 10^-2^** | -0.006  (0.963) | -0.070  (0.552) | -0.138  (0.240) | -0.078  (0.509) | -0.041  (0.729) | -0.075  (0.522) | 0.160  (0.173) | 0.173  (0.141) | **-0.346**  **(0.003)** | 0.061  (0.608) |
| **CX3CR1+ (%)** | 0.081  (0.492) | 0.047  (0.690) | **0.310**  **(0.007)** | 0.200  (0.088) | 0.006  (0.962) | 0.137  (0.245) | -0.024  (0.842) | 0.100  (0.398) | 0.067  (0.569) | 0.108  (0.359) |
| **^¥^CX3CR1+ MFI × 10^-3^** | 0.100  (0.394) | 0.021  (0.859) | 0.217  (0.064) | 0.170  (0.147) | 0.003  (0.977) | 0.022  (0.852) | 0.023  (0.843) | 0.161  (0.170) | -0.027  (0.817) | 0.149  (0.204) |
| **HLA-DR+ (%)** | 0.111  (0.346) | 0.169  (0.151) | 0.145  (0.217) | 0.150  (0.202) | -0.014  (0.903) | 0.128  (0.276) | 0.114  (0.333) | 0.206  (0.079) | 0.115  (0.327) | 0.037  (0.754) |
| **^¥^HLA-DR+ MFI × 10^-2^** | -0.034  (0.772) | -0.033  (0.778) | -0.073  (0.537) | -0.010  (0.931) | 0.024  (0.837) | -0.051  (0.664) | **0.294**  **(0.011)** | 0.216  (0.064) | **-0.329**  **(0.004)** | 0.067  (0.568) |
| **CD11b+ (%)** | -0.029  (0.806) | 0.090  (0.445) | -0.048  (0.684) | -0.012  (0.920) | -0.182  (0.121) | 0.014  (0.903) | -0.093  (0.430) | -0.052  (0.660) | **0.300**  **(0.011)** | -0.074  (0.531) |
| **^¥^CD11b+ MFI × 10^-3^** | -0.023  (0.846) | 0.155  (0.187) | -0.031  (0.794) | -0.025  (0.831) | **-0.260**  **(0.026)** | **0.233**  **(0.046)** | **-0.308**  **(0.008)** | -0.147  (0.211) | **0.300**  **(0.010)** | -0.084  (0.477) |

Notes: CCR2= C-C Motif Chemokine Receptor 2; CCR5= C-C chemokine receptor type 5; CD= cluster of differentiation; CX3CR1= C-X3-C Motif Chemokine Receptor 1; HLA-DR= DR isotype of human leukocyte antigen; hs-CRP, high-sensitivity C-reactive protein; IL-6, interleukin-6; IP-10, IFN-γ-induced protein 10; MFI= mean fluorescent intensity; TNFR, tumor necrosis factor receptor

Bolded values represent statistically significant results

¥ Rescaled

*Spearman Rank correlation coefficient

**Supplementary Table 6e: Non-classical Monocytes**

|  | **All Study Participants (Persons with HIV and without HIV) n=79** | | | | | | | | | |
| --- | --- | --- | --- | --- | --- | --- | --- | --- | --- | --- |
| **Soluble Marker** | **IL-6** | **Hs-CRP** | **TNFR-1** | **TNFR-2** | **CCL2** | **CCL5** | **CX3CL1** | **CXCL10** | **sCD14** | **sCD163** |
|  | ***r**  **(p value)** | ***r**  **(p value)** | ***r**  **(p value)** | ***r**  **(p value)** | ***r**  **(p value)** | ***r**  **(p value)** | ***r**  **(p value)** | ***r**  **(p value)** | ***r**  **(p value)** | ***r**  **(p value)** |
| **Total** | 0.045  (0.701) | 0.104  (0.376) | -0.043  (0.714) | 0.009  (0.937) | 0.078  (0.506) | -0.089  (0.449) | 0.138  (0.239) | **0.240**  **(0.040)** | -0.032  (0.787) | 0.119  (0.313) |
| **CCR2+ (%)** | 0.018  (0.881) | -0.119  (0.312) | 0.028  (0.810) | 0.084  (0.478) | -0.109  (0.356) | 0.008  (0.948) | -0.062  (0.598) | **0.236**  **(0.043)** | 0.177  (0.131) | 0.004  (0.972) |
| **^¥^CCR2+ MFI × 10^-3^** | -0.033  (0.782) | 0.008  (0.948) | -0.043  (0.715) | -0.019  (0.871) | -0.044  (0.711) | -0.088  (0.457) | -0.028  (0.816) | 0.036  (0.764) | -0.115  (0.329) | -0.025  (0.835) |
| **CCR5+ (%)** | -0.061  (0.603) | 0.043  (0.716) | 0.046  (0.698) | 0.101  (0.390) | 0.033  (0.782) | -0.065  (0.584) | -0.146  (0.214) | 0.038  (0.750) | **0.314**  **(0.007)** | -0.041  (0.730) |
| **CD38+ (%)** | -0.179  (0.126) | **-0.232**  **(0.047)** | -0.138  (0.242) | -0.064  (0.590) | 0.005  (0.966) | -0.049  (0.678) | -0.026  (0.828) | **0.250**  **(0.032)** | 0.062  (0.600) | 0.106  (0.370) |
| **^¥^CD38+ MFI × 10^-2^** | -0.157  (0.182) | -0.146  (0.214) | -0.075  (0.524) | 0.018  (0.879) | -0.030  (0.802) | -0.019  (0.872) | -0.190  (0.105) | 0.143  (0.225) | 0.104  (0.376) | 0.021  (0.861) |
| **CD40+ (%)** | 0.202  (0.084) | 0.027  (0.821) | -0.111  (0.344) | -0.046  (0.700) | 0.094  (0.426) | 0.003  (0.981) | 0.096  (0.414) | **0.250**  **(0.032)** | -0.160  (0.173) | **0.280**  **(0.016)** |
| **^¥^CD40+ MFI × 10^-1^** | 0.094  (0.426) | -0.036  (0.759) | -0.159  (0.176) | -0.098  (0.405) | 0.006  (0.958) | -0.001  (0.998) | -0.161  (0.169) | 0.090  (0.447) | -0.116  (0.326) | 0.152  (0.195) |
| **CD163+ (%)** | 0.022  (0.853) | -0.067  (0.570) | 0.091  (0.439) | 0.071  (0.547) | -0.056  (0.633) | -0.122  (0.299) | -0.045  (0.704) | -0.005  (0.967) | **0.267**  **(0.022)** | -0.040  (0.736) |
| **^¥^CD163+ MFI × 10^-2^** | 0.206  (0.079) | 0.095  (0.418) | -0.078  (0.508) | 0.057  (0.626) | 0.113  (0.338) | -0.191  (0.103) | 0.100  (0.395) | 0.215  (0.066) | -0.223  (0.057) | **0.248**  **(0.033)** |
| **CX3CR1+ (%)** | 0.005  (0.969) | -0.003  (0.981) | **0.281**  **(0.016)** | 0.188  (0.110) | -0.056  (0.637) | 0.100  (0.396) | -0.006  (0.959) | 0.081  (0.494) | 0.038  (0.747) | 0.039  (0.743) |
| **^¥^CX3CR1+ MFI × 10^-3^** | -0.021  (0.858) | 0.013  (0.910) | **0.236**  **(0.043)** | **0.230**  **(0.049)** | -0.020  (0.867) | 0.039  (0.743) | -0.008  (0.943) | 0.223  (0.056) | 0.007  (0.951) | 0.134  (0.255) |
| **HLA-DR+ (%)** | **0.235**  **(0.044)** | 0.042  (0.720) | 0.107  (0.362) | 0.068  (0.564) | 0.029  (0.805) | 0.191  (0.103) | 0.012  (0.918) | 0.097  (0.408) | 0.044  (0.711) | -0.006  (0.961) |
| **^¥^HLA-DR+ MFI × 10^-2^** | 0.082  (0.484) | 0.008  (0.947) | 0.008  (0.944) | 0.041  (0.729) | 0.109  (0.354) | -0.022  (0.852) | **0.257**  **(0.027)** | **0.262**  **(0.025)** | **-0.291**  **(0.012)** | 0.114  (0.331) |
| **CD11b+ (%)** | 0.117  (0.319) | -0.069  (0.558) | -0.050  (0.671) | -0.006  (0.963) | -0.004  (0.972) | 0.035  (0.769) | -0.044  (0.708) | 0.120  (0.309) | 0.073  (0.534) | 0.024  (0.837) |
| **^¥^CD11b+ MFI × 10^-3^** | 0.162  (0.168) | 0.049  (0.681) | -0.039  (0.739) | 0.021  (0.858) | -0.105  (0.375) | 0.063  (0.592) | **-0.294**  **(0.011)** | 0.034  (0.773) | 0.207  (0.077) | 0.042  (0.721) |

Notes: CCR2= C-C Motif Chemokine Receptor 2; CCR5= C-C chemokine receptor type 5; CD= cluster of differentiation; CX3CR1= C-X3-C Motif Chemokine Receptor 1; HLA-DR= DR isotype of human leukocyte antigen; hs-CRP, high-sensitivity C-reactive protein; IL-6, interleukin-6; IP-10, IFN-γ-induced protein 10; MFI= mean fluorescent intensity; TNFR, tumor necrosis factor receptor

Bolded values represent statistically significant results

¥ Rescaled

*Spearman Rank correlation coefficient

**Supplementary Tables 7: Correlations Between Soluble Marker and Monocyte Subset and Chemokine Receptor Expression Adjusted for HIV Serostatus**

**Supplementary Table 7a: Classical Monocytes**

|  | **All Study Participants (Persons with HIV and without HIV) n=79** | | | | | | | | | |
| --- | --- | --- | --- | --- | --- | --- | --- | --- | --- | --- |
| **Soluble Marker** | **IL-6** | **Hs-CRP** | **TNFR-1** | **TNFR-2** | **CCL2** | **CCL5** | **CX3CL1** | **CXCL10** | **sCD14** | **sCD163** |
|  | ***r**  **(p value)** | ***r**  **(p value)** | ***r**  **(p value)** | ***r**  **(p value)** | ***r**  **(p value)** | ***r**  **(p value)** | ***r**  **(p value)** | ***r**  **(p value)** | ***r**  **(p value)** | ***r**  **(p value)** |
| **Total** | -0.067  (0.572) | -0.204  (0.083) | 0.006  (0.959) | -0.162  (0.171) | -0.194  (0.100) | 0.030  (0.800) | -0.065  (0.587) | **-0.281**  **(0.016)** | -0.026  (0.828) | **-0.278**  **(0.017)** |
| **CCR2+ (%)** | -0.055  (0.642) | -0.179  (0.129) | -0.027  (0.818) | 0.004  (0.974) | -0.170  (0.149) | 0.195  (0.099) | 0.192  (0.104) | -0.015  (0.901) | -0.167  (0.157) | 0.050  (0.673) |
| **^¥^CCR2+ MFI × 10^-3^** | 0.003  (0.980) | -0.108  (0.364) | -0.145  (0.220) | -0.050  (0.673) | 0.016  (0.893) | 0.069  (0.563) | **0.235**  **(0.045)** | -0.032  (0.785) | **-0.299**  **(0.010)** | 0.059  (0.619) |
| **CCR5+ (%)** | -0.046  (0.700) | -0.055  (0.642) | -0.069  (0.560) | -0.029  (0.809) | 0.051  (0.670) | -0.186  (0.114) | -0.019  (0.871) | 0.072  (0.544) | 0.155  (0.191) | -0.031  (0.796) |
| **CD38+ (%)** | -0.142  (0.232) | **-0.315**  **(0.007)** | -0.012  (0.919) | -0.064  (0.592) | -0.161  (0.174) | -0.110  (0.354) | 0.090  (0.449) | 0.096  (0.417) | 0.147  (0.215) | -0.068  (0.569) |
| **^¥^CD38+ MFI × 10^-2^** | 0.065  (0.583) | -0.083  (0.484) | -0.069  (0.562) | -0.037  (0.753) | 0.105  (0.378) | -0.024  (0.842) | 0.085  (0.477) | 0.174  (0.140) | -0.032  (0.791) | -0.009  (0.938) |
| **CD40+ (%)** | 0.085  (0.473) | 0.076  (0.520) | 0.085  (0.476) | 0.111  (0.348) | 0.019  (0.870) | 0.174  (0.141) | 0.123  (0.298) | 0.127  (0.283) | -0.044  (0.709) | -0.003  (0.978) |
| **^¥^CD40+ MFI × 10^-1^** | -0.070  (0.559) | -0.048  (0.686) | -0.020  (0.868) | -0.037  (0.755) | -0.088  (0.460) | 0.216  (0.066) | -0.170  (0.150) | -0.020  (0.865) | 0.044  (0.709) | -0.157  (0.184) |
| **CD163+ (%)** | -0.187  (0.113) | -0.179  (0.131) | 0.096  (0.420) | -0.031  (0.794) | -0.129  (0.276) | 0.047  (0.695) | 0.091  (0.496) | 0.073  (0.541) | 0.080  (0.502) | **-0.233**  **(0.047)** |
| **^¥^CD163+ MFI × 10^-2^** | -0.122  (0.305) | -0.175  (0.138) | -0.155  (0.189) | -0.110  (0.355) | -0.140  (0.238) | 0.073  (0.537) | 0.179  (0.130) | 0.031  (0.791) | **-0.285**  **(0.014)** | -0.043  (0.715) |
| **CX3CR1+ (%)** | -0.030  (0.799) | -0.183  (0.120) | -0.040  (0.734) | -0.040  (0.737) | -0.038  (0.748) | 0.092  (0.439) | 0.202  (0.087) | 0.153  (0.197) | -0.023  (0.849) | 0.100  (0.400) |
| **^¥^CX3CR1+ MFI × 10^-3^** | -0.012  (0.919) | -0.057  (0.631) | -0.023  (0.847) | 0.067  (0.575) | -0.030  (0.800) | -0.034  (0.778) | 0.176  (0.136) | 0.013  (0.914) | -0.165  (0.163) | 0.088  (0.457) |
| **HLA-DR+ (%)** | 0.112  (0.344) | -0.145  (0.221) | -0.116  (0.329) | -0.114  (0.336) | 0.080  (0.501) | 0.016  (0.894) | 0.203  (0.085) | 0.162  (0.171) | -0.141  (0.234) | 0.078  (0.510) |
| **^¥^HLA-DR+ MFI × 10^-2^** | -0.021  (0.859) | -0.081  (0.493) | -0.137  (0.249) | -0.019  (0.875) | -0.035  (0.768) | -0.005  (0.969) | 0.190  (0.107) | 0.030  (0.803) | -0.198  (0.093) | 0.117  (0.324) |
| **CD11b+ (%)** | -0.014  (0.907) | 0.067  (0.571) | 0.174  (0.141) | **0.240**  **(0.041)** | 0.078  (0.513) | -0.052  (0.661) | -0.035  (0.769) | 0.045  (0.707) | 0.018  (0.882) | 0.038  (0.748) |
| **^¥^CD11b+ MFI × 10^-3^** | -0.172  (0.145) | -0.012  (0.919) | -0.125  (0.291) | -0.143  (0.226) | -0.009  (0.938) | 0.161  (0.175) | -0.147  (0.215) | -0.074  (0.534) | 0.090  (0.451) | -0.093  (0.434) |

Notes: CCR2= C-C Motif Chemokine Receptor 2; CCR5= C-C chemokine receptor type 5; CD= cluster of differentiation; CX3CR1= C-X3-C Motif Chemokine Receptor 1; HLA-DR= DR isotype of human leukocyte antigen; hs-CRP, high-sensitivity C-reactive protein; IL-6, interleukin-6; IP-10, IFN-γ-induced protein 10; MFI= mean fluorescent intensity; TNFR, tumor necrosis factor receptor

Bolded values represent statistically significant results

¥ Rescaled

*Partial Correlation Coefficient controlling for serostatus

**Supplementary Table 7b: CD16dim Monocytes**

|  | **All Study Participants (Persons with HIV and without HIV) n=79** | | | | | | | | | |
| --- | --- | --- | --- | --- | --- | --- | --- | --- | --- | --- |
| **Soluble Marker** | **IL-6** | **Hs-CRP** | **TNFR-1** | **TNFR-2** | **CCL2** | **CCL5** | **CX3CL1** | **CXCL10** | **sCD14** | **sCD163** |
|  | ***r**  **(p value)** | ***r**  **(p value)** | ***r**  **(p value)** | ***r**  **(p value)** | ***r**  **(p value)** | ***r**  **(p value)** | ***r**  **(p value)** | ***r**  **(p value)** | ***r**  **(p value)** | ***r**  **(p value)** |
| **Total** | -0.003  (0.979) | -0.118  (0.319) | 0.079  (0.504) | 0.018  (0.883) | -0.097  (0.415) | 0.062  (0.602) | 0.004  (0.976) | -0.068  (0.566) | **0.269**  **(0.021)** | -0.140  (0.238) |
| **CCR2+ (%)** | -0.053  (0.658) | -0.156  (0.186) | -0.064  (0.592) | -0.015  (0.901) | -0.021  (0.857) | 0.191  (0.106) | **0.248**  **(0.034)** | 0.052  (0.664) | -0.185  (0.118) | 0.079  (0.507) |
| **^¥^CCR2+ MFI × 10^-3^** | -0.002  (0.989) | -0.095  (0.425) | -0.164  (0.166) | -0.072  (0.548) | 0.015  (0.901) | 0.044  (0.711) | **0.272**  **(0.020)** | -0.039  (0.740) | **-0.347**  **(0.003)** | 0.055  (0.643) |
| **CCR5+ (%)** | -0.042  (0.724) | -0.046  (0.701) | -0.007  (0.954) | 0.011  (0.927) | 0.211  (0.074) | -0.185  (0.117) | 0.043  (0.718) | **0.271**  **(0.021)** | 0.135  (0.256) | -0.040  (0.735) |
| **CD38+ (%)** | -0.144  (0.225) | -0.169  (0.153) | -0.032  (0.791) | -0.026  (0.829) | 0.043  (0.719) | -0.067  (0.572) | 0.193  (0.102) | 0.156  (0.188) | 0.044  (0.709) | -0.011  (0.923) |
| **^¥^CD38+ MFI × 10^-2^** | **0.515**  **(0.001)** | -0.037  (0.756) | -0.155  (0.191) | -0.090  (0.451) | -0.051  (0.665) | -0.164  (0.167) | -0.084  (0.478) | -0.037  (0.757) | 0.169  (0.153) | -0.111  (0.350) |
| **CD40+ (%)** | 0.034  (0.773) | 0.040  (0.734) | 0.011  (0.924) | 0.027  (0.822) | 0.142  (0.231) | -0.130  (0.274) | 0.161  (0.173) | 0.132  (0.265) | -0.017  (0.887) | 0.057  (0.633) |
| **^¥^CD40+ MFI × 10^-1^** | 0.199  (0.092) | 0.137  (0.248) | **0.274**  **(0.019)** | 0.161  (0.174) | **0.647**  **(0.001)** | -0.128  (0.280) | 0.167  (0.158) | **0.786**  **(0.001)** | 0.175  (0.138) | -0.061  (0.605) |
| **CD163+ (%)** | -0.225  (0.055) | -0.099  (0.405) | -0.016  (0.890) | -0.035  (0.772) | 0.034  (0.776) | -0.094  (0.427) | 0.134  (0.258) | 0.069  (0.563) | -0.154  (0.192) | 0.034  (0.776) |
| **^¥^CD163+ MFI × 10^-2^** | 0.015  (0.899) | -0.070  (0.558) | **0.254**  **(0.030)** | 0.161  (0.174) | 0.102  (0.391) | -0.161  (0.174) | **0.495**  **(0.001)** | **0.290**  **(0.013)** | -0.147  (0.215) | -0.063  (0.597) |
| **CX3CR1+ (%)** | -0.153  (0.196) | **-0.303**  **(0.009)** | -0.037  (0.755) | -0.038  (0.749) | -0.116  (0.330) | 0.081  (0.494) | 0.139  (0.241) | 0.041  (0.732) | 0.050  (0.676) | 0.085  (0.476) |
| **^¥^CX3CR1+ MFI × 10^-3^** | -0.100  (0.401) | -0.147  (0.215) | -0.084  (0.480) | -0.001  (0.991) | -0.084  (0.480) | -0.027  (0.822) | 0.112  (0.347) | -0.074  (0.536) | -0.192  (0.103) | 0.059  (0.618) |
| **HLA-DR+ (%)** | 0.081  (0.497) | -0.086  (0.470) | -0.116  (0.330) | -0.077  (0.518) | 0.191  (0.106) | 0.021  (0.857) | 0.201  (0.087) | 0.169  (0.154) | -0.196  (0.097) | 0.069  (0.560) |
| **^¥^HLA-DR+ MFI × 10^-2^** | -0.025  (0.837) | -0.086  (0.471) | -0.119  (0.318) | -0.016  (0.894) | -0.025  (0.832) | 0.036  (0.760) | 0.174  (0.141) | 0.017  (0.886) | -0.162  (0.172) | 0.104  (0.382) |
| **CD11b+ (%)** | 0.037  (0.757) | 0.032  (0.785) | -0.080  (0.502) | 0.033  (0.781) | 0.023  (0.845) | -0.186  (0.115) | 0.124  (0.297) | 0.058  (0.628) | -0.167  (0.158) | -0.030  (0.802) |
| **^¥^CD11b+ MFI × 10^-3^** | -0.014  (0.907) | -0.031  (0.797) | -0.158  (0.182) | -0.176  (0.136) | -0.011  (0.927) | 0.146  (0.218) | -0.163  (0.167) | -0.063  (0.595) | 0.128  (0.281) | -0.133  (0.260) |

Notes: CCR2= C-C Motif Chemokine Receptor 2; CCR5= C-C chemokine receptor type 5; CD= cluster of differentiation; CX3CR1= C-X3-C Motif Chemokine Receptor 1; HLA-DR= DR isotype of human leukocyte antigen; hs-CRP, high-sensitivity C-reactive protein; IL-6, interleukin-6; IP-10, IFN-γ-induced protein 10; MFI= mean fluorescent intensity; TNFR, tumor necrosis factor receptor

Bolded values represent statistically significant results

¥ Rescaled

*Partial Correlation Coefficient controlling for serostatus

**Supplementary Table 7c: CD16neg Monocytes**

|  | **All Study Participants (Persons with HIV and without HIV) n=79** | | | | | | | | | |
| --- | --- | --- | --- | --- | --- | --- | --- | --- | --- | --- |
| **Soluble Marker** | **IL-6** | **Hs-CRP** | **TNFR-1** | **TNFR-2** | **CCL2** | **CCL5** | **CX3CL1** | **CXCL10** | **sCD14** | **sCD163** |
|  | ***r**  **(p value)** | ***r**  **(p value)** | ***r**  **(p value)** | ***r**  **(p value)** | ***r**  **(p value)** | ***r**  **(p value)** | ***r**  **(p value)** | ***r**  **(p value)** | ***r**  **(p value)** | ***r**  **(p value)** |
| **Total** | -0. 017  (0.886) | 0.008  (0.948) | -0.127  (0.285) | -0.133  (0.261) | -0.106  (0.372) | 0.016  (0.892) | -0.166  (0.159) | -0.167  (0.158) | **-0.234**  **(0.047)** | -0.038  (0.752) |
| **CCR2+ (%)** | 0.019  (0.872) | -0.028  (0.817) | -0.068  (0.565) | 0.007  (0.954) | 0.005  (0.965) | 0.192  (0.103) | 0.173  (0.142) | 0.005  (0.968) | -0.185  (0.117) | 0.131  (0.268) |
| **^¥^CCR2+ MFI × 10^-3^** | 0.021  (0.860) | -0.107  (0.366) | -0.186  (0.115) | -0.104  (0.381) | -0.016  (0.893) | 0.057  (0.634) | 0.214  (0.069) | -0.036  (0.760) | **-0.366**  **(0.001)** | 0.073  (0.541) |
| **CCR5+ (%)** | **0.269**  **(0.021)** | 0.042  (0.725) | **0.272**  **(0.020)** | 0.120  (0.314) | **0.584**  **(0.001)** | 0.017  (0.888) | 0.182  (0.124) | **0.642**  **(0.001)** | 0.165  (0.163) | -0.079  (0.507) |
| **CD38+ (%)** | -0.021  (0.860) | -0.086  (0.470) | -0.044  (0.711) | 0.001  (0.994) | 0.109  (0.360) | -0.088  (0.459) | **0.270**  **(0.021)** | 0.218  (0.064) | -0.059  (0.617) | 0.106  (0.372) |
| **^¥^CD38+ MFI × 10^-2^** | **0.551**  **(0.001)** | -0.036  (0.760) | -0.141  (0.233) | -0.080  (0.502) | -0.024  (0.842) | -0.165  (0.162) | -0.059  (0.621) | -0.009  (0.939) | 0.171  (0.147) | -0.096  (0.421) |
| **CD40+ (%)** | 0.143  (0.226) | 0.034  (0.775) | 0.027  (0.820) | 0.118  (0.320) | 0.156  (0.188) | -0.006  (0.962) | **0.237**  **(0.043)** | 0.160  (0.175) | -0.190  (0.106) | 0.101  (0.397) |
| **^¥^CD40+ MFI × 10^-1^** | -0.065  (0.583) | -0.068  (0.570) | -0.138  (0.245) | -0.045  (0.703) | -0.007  (0.954) | 0.069  (0.560) | -0.006  (0.958) | -0.006  (0.962) | -0.202  (0.087) | -0.027  (0.820) |
| **CD163+ (%)** | -0.164  (0.166) | -0.223  (0.058) | -0.203  (0.086) | -0.121  (0.309) | -0.150  (0.206) | 0.139  (0.241) | -0.025  (0.834) | -0.071  (0.553) | -0.205  (0.082) | 0.038  (0.747) |
| **^¥^CD163+ MFI × 10^-2^** | -0.134  (0.260) | -0.181  (0.126) | **-0. 275**  **(0.018)** | -0.149  (0.207) | -0.134  (0.257) | 0.175  (0.139) | 0.008  (0.949) | -0.057  (0.635) | **-0.295**  **(0.011)** | -0.047  (0.695) |
| **CX3CR1+ (%)** | -0.060  (0.615) | -0.121  (0.308) | -0.016  (0.894) | 0.046  (0.696) | 0.042  (0.722) | 0.040  (0.735) | 0.134  (0.258) | 0.020  (0.866) | -0.122  (0.305) | 0.173  (0.144) |
| **^¥^CX3CR1+ MFI × 10^-3^** | 0.044  (0.712) | 0.002  (0.988) | 0.001  (0.997) | 0.082  (0.492) | 0.101  (0.396) | 0.014  (0.904) | 0.187  (0.114) | -0.012  (0.923) | -0.220  (0.061) | 0.112  (0.347) |
| **HLA-DR+ (%)** | 0.053  (0.657) | -0.125  (0.293) | -0.155  (0.192) | -0.131  (0.270) | 0.154  (0.193) | 0.020  (0.864) | **0.247**  **(0.035)** | 0.160  (0.177) | -0.187  (0.114) | 0.053  (0.659) |
| **^¥^HLA-DR+ MFI × 10^-2^** | -0.010  (0.932) | -0.047  (0.690) | -0.140  (0.237) | -0.037  (0.754) | -0.004  (0.976) | -0.007  (0.950) | 0.182  (0.122) | 0.019  (0.872) | **-0.266**  **(0.023)** | 0.124  (0.298) |
| **CD11b+ (%)** | 0.067  (0.572) | -0.016  (0.895) | 0.148  (0.210) | 0.096  (0.421) | -0.007  (0.951) | -0.196  (0.096) | -0.067  (0.573) | 0.032  (0.787) | 0.136  (0.251) | 0.058  (0.624) |
| **^¥^CD11b+ MFI × 10^-3^** | -0.008  (0.946) | 0.032  (0.790) | -0.106  (0.372) | -0.126  (0.287) | 0.030  (0.803) | 0.017  (0.887) | -0.106  (0.370) | -0.031  (0.797) | 0.117  (0.324) | -0.120  (0.311) |

Notes: CCR2= C-C Motif Chemokine Receptor 2; CCR5= C-C chemokine receptor type 5; CD= cluster of differentiation; CX3CR1= C-X3-C Motif Chemokine Receptor 1; HLA-DR= DR isotype of human leukocyte antigen; hs-CRP, high-sensitivity C-reactive protein; IL-6, interleukin-6; IP-10, IFN-γ-induced protein 10; MFI= mean fluorescent intensity; TNFR, tumor necrosis factor receptor

Bolded values represent statistically significant results

¥ Rescaled

*Partial Correlation Coefficient controlling for serostatus

**Supplementary Table 7d: Intermediate Monocytes**

|  | **All Study Participants (Persons with HIV and without HIV) n=79** | | | | | | | | | |
| --- | --- | --- | --- | --- | --- | --- | --- | --- | --- | --- |
| **Soluble Marker** | **IL-6** | **Hs-CRP** | **TNFR-1** | **TNFR-2** | **CCL2** | **CCL5** | **CX3CL1** | **CXCL10** | **sCD14** | **sCD163** |
|  | ***r**  **(p value)** | ***r**  **(p value)** | ***r**  **(p value)** | ***r**  **(p value)** | ***r**  **(p value)** | ***r**  **(p value)** | ***r**  **(p value)** | ***r**  **(p value)** | ***r**  **(p value)** | ***r**  **(p value)** |
| **Total** | **0.254**  **(0.030)** | 0.068  (0.570) | 0.176  (0.136) | **0.273**  **(0.019)** | **0.316**  **(0.006)** | -0.137  (0.249) | 0.146  (0.217) | **0.495**  **(0.001)** | 0.164  (0.167) | **0.369**  **(0.001)** |
| **CCR2+ (%)** | -0.019  (0.874) | -0.102  (0.393) | 0.016  (0.891) | 0.155  (0.192) | -0.106  (0.372) | -0.019  (0.871) | 0.064  (0.593) | 0.100  (0.400) | -0.063  (0.595) | 0.101  (0.393) |
| **^¥^CCR2+ MFI × 10^-3^** | -0.067  (0.575) | -0.090  (0.450) | -0.154  (0.194) | -0.046  (0.701) | -0.049  (0.680) | -0.049  (0.683) | 0.112  (0.345) | 0.007  (0.955) | -0.228  (0.052) | 0.096  (0.418) |
| **CCR5+ (%)** | -0.003  (0.983) | -0.092  (0.439) | 0.031  (0.796) | 0.052  (0.665) | 0.024  (0.838) | -0.056  (0.640) | 0.009  (0.937) | 0.054  (0.651) | **0.258**  **(0.028)** | -0.093  (0.434) |
| **CD38+ (%)** | -0.043  (0.718) | -0.060  (0.616) | 0.099  (0.402) | 0.197  (0.095) | 0.061  (0.610) | -0.120  (0.314) | 0.007  (0.950) | **0.260**  **(0.026)** | 0.170  (0.149) | 0.081  (0.494) |
| **^¥^CD38+ MFI × 10^-2^** | -0.013  (0.912) | -0.012  (0.919) | 0.065  (0.587) | **0.230**  **(0.050)** | 0.074  (0.531) | -0.119  (0.315) | -0.017  (0.890) | **0.239**  **(0.042)** | 0.071  (0.549) | 0.140  (0.238) |
| **CD40+ (%)** | 0.067  (0.572) | 0.003  (0.981) | 0.041  (0.730) | 0.124  (0.297) | 0.001  (0.995) | -0.022  (0.852) | 0.001  (0.993) | 0.194  (0.100) | 0.105  (0.378) | 0.119  (0.314) |
| **^¥^CD40+ MFI × 10^-1^** | -0.068  (0.567) | -0.103  (0.385) | 0.002  (0.984) | 0.078  (0.512) | -0.096  (0.420) | -0.064  (0.592) | -0.192  (0.104) | 0.062  (0.603) | 0.079  (0.505) | -0.011  (0.928) |
| **CD163+ (%)** | -0.173  (0.143) | -0.095  (0.424) | 0.057  (0.633) | -0.045  (0.707) | -0.074  (0.532) | 0.061  (0.611) | 0.013  (0.912) | 0.122  (0.302) | 0.086  (0.472) | -0.160  (0.175) |
| **^¥^CD163+ MFI × 10^-2^** | -0.040  (0.735) | -0.116  (0.329) | -0.227  (0.054) | -0.128  (0.282) | -0.112  (0.346) | 0.036  (0.764) | 0.061  (0.609) | 0.020  (0.869) | **-0.352**  **(0.002)** | 0.102  (0.392) |
| **CX3CR1+ (%)** | 0.066  (0.580) | 0.046  (0.698) | 0.074  (0.531) | 0.095  (0.422) | -0.076  (0.523) | 0.029  (0.804) | 0.008  (0.949) | -0.196  (0.096) | -0.144  (0.225) | 0.180  (0.127) |
| **^¥^CX3CR1+ MFI × 10^-3^** | 0.113  (0.343) | 0.161  (0.175) | 0.114  (0.337) | 0.131  (0.269) | 0.056  (0.639) | -0.012  (0.919) | 0.146  (0.218) | -0.074  (0.537) | -0.090  (0.450) | 0.201  (0.088) |
| **HLA-DR+ (%)** | 0.165  (0.164) | 0.123  (0.302) | 0.003  (0.983) | 0.036  (0.764) | 0.031  (0.792) | 0.071  (0.553) | 0.089  (0.453) | 0.139  (0.242) | -0.054  (0.649) | 0.129  (0.275) |
| **^¥^HLA-DR+ MFI × 10^-2^** | -0.003  (0.979) | -0.069  (0.564) | -0.180  (0.127) | 0.008  (0.945) | -0.046  (0.697) | 0.088  (0.459) | 0.191  (0.106) | 0.065  (0.583) | **-0.284**  **(0.015)** | **0.245**  **(0.037)** |
| **CD11b+ (%)** | 0.001  (0.998) | 0.055  (0.646) | -0.213  (0.071) | 0.006  (0.961) | -0.075  (0.529) | 0.039  (0.742) | -0.132  (0.267) | 0.100  (0.400) | -0.014  (0.906) | 0.113  (0.340) |
| **^¥^CD11b+ MFI × 10^-3^** | -0.107  (0.365) | 0.118  (0.319) | -0.035  (0.768) | -0.017  (0.886) | -0.024  (0.840) | **0.254**  **(0.030)** | **-0.248**  **(0.034)** | 0.027  (0.822) | **0.267**  **(0.022)** | -0.146  (0.219) |

Notes: CCR2= C-C Motif Chemokine Receptor 2; CCR5= C-C chemokine receptor type 5; CD= cluster of differentiation; CX3CR1= C-X3-C Motif Chemokine Receptor 1; HLA-DR= DR isotype of human leukocyte antigen; hs-CRP, high-sensitivity C-reactive protein; IL-6, interleukin-6; IP-10, IFN-γ-induced protein 10; MFI= mean fluorescent intensity; TNFR, tumor necrosis factor receptor

Bolded values represent statistically significant results

¥ Rescaled

*Partial Correlation Coefficient controlling for serostatus

**Supplementary Table 7e: Non-classical Monocytes**

|  | **All Study Participants (Persons with HIV and without HIV) n=79** | | | | | | | | | |
| --- | --- | --- | --- | --- | --- | --- | --- | --- | --- | --- |
| **Soluble Marker** | **IL-6** | **Hs-CRP** | **TNFR-1** | **TNFR-2** | **CCL2** | **CCL5** | **CX3CL1** | **CXCL10** | **sCD14** | **sCD163** |
|  | ***r**  **(p value)** | ***r**  **(p value)** | ***r**  **(p value)** | ***r**  **(p value)** | ***r**  **(p value)** | ***r**  **(p value)** | ***r**  **(p value)** | ***r**  **(p value)** | ***r**  **(p value)** | ***r**  **(p value)** |
| **Total** | 0. 021  (0.857) | 0.206  (0.081) | -0.054  (0.652) | 0.118  (0.319) | 0.153  (0.197) | -0.008  (0.945) | 0.064  (0.593) | 0.198  (0.094) | -0.030  (0.798) | **0.277**  **(0.018)** |
| **CCR2+ (%)** | 0.127  (0.285) | -0.042  (0.723) | 0.053  (0.655) | -0.030  (0.801) | -0.013  (0.910) | -0.011  (0.928) | -0.133  (0.262) | -0.041  (0.728) | 0.139  (0.241) | -0.023  (0.848) |
| **^¥^CCR2+ MFI × 10^-3^** | 0.075  (0.531) | -0.098  (0.407) | -0.039  (0.746) | -0.077  (0.516) | -0.051  (0.670) | 0.022  (0.855) | -0.062  (0.602) | -0.055  (0.647) | 0.025  (0.831) | -0.025  (0.831) |
| **CCR5+ (%)** | 0.018  (0.878) | -0.071  (0.553) | -0.027  (0.820) | -0.037  (0.756) | 0.014  (0.905) | -0.089  (0.452) | -0.132  (0.265) | -0.045  (0.706) | 0.191  (0.105) | -0.070  (0.557) |
| **CD38+ (%)** | -0.074  (0.536) | -0.118  (0.321) | -0.022  (0.855) | 0.010  (0.933) | 0.040  (0.736) | -0.073  (0.538) | -0.087  (0.463) | 0.164  (0.166) | 0.108  (0.363) | -0.011  (0.925) |
| **^¥^CD38+ MFI × 10^-2^** | -0.103  (0.387) | -0.055  (0.647) | -0.032  (0.787) | -0.031  (0.798) | -0.043  (0.719) | 0.012  (0.921) | -0.129  (0.275) | 0.015  (0.903) | 0.093  (0.432) | -0.003  (0.982) |
| **CD40+ (%)** | 0.218  (0.064) | 0.002  (0.988) | -0.028  (0.813) | 0.136  (0.251) | 0.062  (0.600) | -0.016  (0.895) | 0.115  (0.333) | **0.265**  **(0.024)** | -0.173  (0.143) | **0.255**  **(0.029)** |
| **^¥^CD40+ MFI × 10^-1^** | 0.099  (0.404) | -0.077  (0.517) | -0.093  (0.432) | 0.031  (0.797) | -0.045  (0.703) | 0.015  (0.901) | -0.099  (0.404) | 0.155  (0.191) | -0.166  (0.160) | 0.146  (0.218) |
| **CD163+ (%)** | 0.005  (0.965) | -0.058  (0.624) | 0. 113  (0.341) | 0.093  (0.434) | 0.047  (0.693) | 0.032  (0.787) | -0.045  (0.704) | 0.113  (0.342) | 0.209  (0.076) | -0.046  (0.699) |
| **^¥^CD163+ MFI × 10^-2^** | 0.205  (0.081) | -0.009  (0.938) | -0.079  (0.507) | 0.055  (0.645) | -0.014  (0.905) | -0.124  (0.295) | 0.088  (0.460) | 0.105  (0.375) | -0.212  (0.071) | **0.271**  **(0.021)** |
| **CX3CR1+ (%)** | 0.038  (0.747) | 0.133  (0.262) | 0.092  (0.437) | 0.114  (0.337) | 0.112  (0.344) | -0.024  (0.842) | 0.124  (0.296) | 0.087  (0.463) | -0.168  (0.154) | 0.185  (0.117) |
| **^¥^CX3CR1+ MFI × 10^-3^** | -0.054  (0.649) | 0.101  (0.393) | 0.094  (0.430) | 0.130  (0.274) | 0.090  (0.449) | -0.106  (0.373) | 0.086  (0.468) | 0.062  (0.605) | 0.037  (0.755) | 0.077  (0.516) |
| **HLA-DR+ (%)** | 0.193  (0.101) | 0.063  (0.595) | 0.058  (0.623) | 0.082  (0.493) | -0.005  (0.967) | 0.134  (0.258) | 0.001  (0.994) | 0.007  (0.950) | 0.049  (0.678) | 0.104  (0.379) |
| **^¥^HLA-DR+ MFI × 10^-2^** | 0.113  (0.343) | -0.048  (0.685) | -0.146  (0.219) | 0.026  (0.829) | -0.022  (0.853) | 0.096  (0.419) | 0.198  (0.094) | 0.093  (0.435) | **-0.290**  **(0.013)** | **0.283**  **(0.015)** |
| **CD11b+ (%)** | 0.180  (0.127) | -0.040  (0.737) | -0.070  (0.559) | 0.036  (0.761) | -0.013  (0.911) | 0.066  (0.581) | -0.019  (0.874) | 0.167  (0.158) | -0.004  (0.973) | 0.110  (0.354) |
| **^¥^CD11b+ MFI × 10^-3^** | 0.138  (0.245) | 0.055  (0.646) | 0.055  (0.642) | 0.071  (0.548) | -0.048  (0.686) | 0.161  (0.173) | **-0.283**  **(0.015)** | -0.008  (0.944) | 0.243  (0.038) | -0.093  (0.435) |

Notes: CCR2= C-C Motif Chemokine Receptor 2; CCR5= C-C chemokine receptor type 5; CD= cluster of differentiation; CX3CR1= C-X3-C Motif Chemokine Receptor 1; HLA-DR= DR isotype of human leukocyte antigen; hs-CRP, high-sensitivity C-reactive protein; IL-6, interleukin-6; IP-10, IFN-γ-induced protein 10; MFI= mean fluorescent intensity; TNFR, tumor necrosis factor receptor

Bolded values represent statistically significant results

*Partial Correlation Coefficient controlling for serostatus

**__________**

**Supplemental Figure 3a: Correlations between CD4+ TCM subsets and associated cell surface markers and monocyte subsets and associated cell surface markers among all study participants** Clustered heatmap of statistically significant Pearson correlations between CD4+ TCM subsets and monocyte subsets. Heatmap tiles show Pearson correlation coefficients (r) for associations between cellular markers (rows) and soluble markers (columns). Correlation values were standardized using z-scores across each row. Colors indicate the direction and magnitude of r (red = positive, blue = negative, white ≈ 0). Rows and columns are hierarchically clustered using correlation distance (1 − r) to group markers with similar correlation profiles; dendrograms depict cluster structure.

**Supplemental Figure 3b: Correlations between CD4+ TEM subsets and associated cell surface markers and monocyte subsets and associated cell surface markers among all study participants.** Clustered heatmap of statistically significant Pearson correlations between CD4+ TEM subsets and monocyte subsets. Heatmap tiles show Pearson correlation coefficients (r) for associations between cellular markers (rows) and soluble markers (columns). Correlation values were standardized using z-scores across each row. Colors indicate the direction and magnitude of r (red = positive, blue = negative, white ≈ 0). Rows and columns are hierarchically clustered using correlation distance (1 − r) to group markers with similar correlation profiles; dendrograms depict cluster structure.

**Supplemental Figure 3c: Correlations between CD4+ TEMRA subsets and associated cell surface markers and monocyte subsets and associated cell surface markers among all study participants.** Clustered heatmap of statistically significant Pearson correlations between CD4+ TEMRA subsets and monocyte subsets. Heatmap tiles show Pearson correlation coefficients (r) for associations between cellular markers (rows) and soluble markers (columns). Correlation values were standardized using z-scores across each row. Colors indicate the direction and magnitude of r (red = positive, blue = negative, white ≈ 0). Rows and columns are hierarchically clustered using correlation distance (1 − r) to group markers with similar correlation profiles; dendrograms depict cluster structure.

**Supplemental Figure 3d: Correlations between CD8+ TCM subsets and associated cell surface markers and monocyte subsets and associated cell surface markers among all study participants.** Clustered heatmap of statistically significant Pearson correlations between CD8+ TCM subsets and monocyte subsets. Heatmap tiles show Pearson correlation coefficients (r) for associations between cellular markers (rows) and soluble markers (columns). Correlation values were standardized using z-scores across each row. Colors indicate the direction and magnitude of r (red = positive, blue = negative, white ≈ 0). Rows and columns are hierarchically clustered using correlation distance (1 − r) to group markers with similar correlation profiles; dendrograms depict cluster structure.

**Supplemental Figure 3e: Correlations between CD8+ TEM subsets and associated cell surface markers and monocyte subsets and associated cell surface markers among all study participants.** Clustered heatmap of statistically significant Pearson correlations between CD8+ TCM subsets and monocyte subsets. Heatmap tiles show Pearson correlation coefficients (r) for associations between cellular markers (rows) and soluble markers (columns). Correlation values were standardized using z-scores across each row. Colors indicate the direction and magnitude of r (red = positive, blue = negative, white ≈ 0). Rows and columns are hierarchically clustered using correlation distance (1 − r) to group markers with similar correlation profiles; dendrograms depict cluster structure.

**Supplemental Figure 3f: Correlations between CD8+ TEMRA subsets and associated cell surface markers and monocyte subsets and associated cell surface markers among all study participants.** Clustered heatmap of statistically significant Pearson correlations between CD8+ TEMRA subsets and monocyte subsets. Heatmap tiles show Pearson correlation coefficients (r) for associations between cellular markers (rows) and soluble markers (columns). Correlation values were standardized using z-scores across each row. Colors indicate the direction and magnitude of r (red = positive, blue = negative, white ≈ 0). Rows and columns are hierarchically clustered using correlation distance (1 − r) to group markers with similar correlation profiles; dendrograms depict cluster structure.

Notes: CCR2= C-C Motif Chemokine Receptor 2; CCR5= C-C chemokine receptor type 5; CD= cluster of differentiation; CXCL= C-X-C Motif Chemokine Ligand; CX3CR1= C-X3-C Motif Chemokine Receptor 1; DR= DR isotype of human leukocyte antigen; hs-CRP, high-sensitivity C-reactive protein; IL-6, interleukin-6; MFI= mean fluorescent intensity; TNFR, tumor necrosis factor receptor, NCM = nonclassical monocytes, CD16neg = CD16 negative monocytes, IM = intermediate monocytes, CD16dim = CD16 dim intermediate/inflammatory monocytes, CM = classical monocytes
